# Rewriting the phase diagram of a diamagnetic liquid crystal by a magnetic field

**DOI:** 10.1038/s41467-018-06976-7

**Published:** 2018-10-25

**Authors:** Fatin Hajjaj, Takashi Kajitani, Hiroyuki Ohsumi, Yoshikazu Tanaka, Kenichi Kato, Masaki Takata, Hideaki Kitazawa, Taka-hisa Arima, Takuzo Aida, Takanori Fukushima

**Affiliations:** 1 Laboratory for Chemistry and Life Science, Institute of Innovative Research, Tokyo Institute of Technology, 4259 Nagatsuta, Midori-ku, Yokohama, 226-8503 Japan; 2 RIKEN SPring-8 Center, 1-1-1 Kouto, Sayo, Hyogo 679-5148 Japan; 3 Institute of Multidisciplinary Research for Advanced Materials, Tohoku University, 2-1-1 Katahira, Aoba-ku, Sendai, 980-8577 Japan; 4 National Institute for Materials Science, 1-2-1 Sengen, Tsukuba, Ibaraki 305-0047 Japan; 5 Department of Advanced Materials Science, The University of Tokyo, 5-1-5 Kashiwanoha, Kashiwa, Chiba 277-8561 Japan; 6 RIKEN Center for Emergent Matter Science (CEMS), 2-1 Hirosawa, Wako, Saitama 351-0198 Japan; 7 Department of Chemistry and Biotechnology, School of Engineering, The University of Tokyo, 7-3-1 Hongo, Bunkyo-ku, Tokyo, 113-8656 Japan

## Abstract

Magnetic fields have been considered to only interact with organic materials non-destructively, leaving their fundamental structures unaffected, even when a strong magnetic field generated from a superconducting magnet is applied. Here we report an unprecedented observation that a liquid-crystalline mesophase of a diamagnetic molecular assembly with an orthorhombic or a cubic structure is formed selectively in the absence or presence of a strong magnetic field. The constituent molecule is a triphenylene derivative carrying six imidazolium bromide-terminated alkyl side chains and exhibits a cubic, orthorhombic, or hexagonal columnar mesophase when complexed with an appropriate amount of lanthanum(III) bromide. Thermal processing of the La^3+^-containing liquid-crystalline assembly in the presence of a 10-tesla magnetic field resulted in a phase diagram, in which the orthorhombic phase is completely replaced with the cubic phase. The discovery of this magneto-induced phase-selection offers an insight into the interactions between magnetic fields and organic material.

## Abstract (web summary)

Magnetically induced phase behaviour in a soft matter system is of potential interest for magneto-responsive compounds. Here the authors fabricate a discotic ionic liquid crystalline hybrid material which can be switched from orthorhombic to cubic phase in the absence or presence of a strong magnetic field.

## Introduction

The interactions between fields and matter represent not only a principal topic in fundamental science but also form the basis of functionality. Magnetic fields, for example, play a pivotal role in switching the physical properties of materials associated with spin^1,2^. In addition, magnetic fields can non-destructively orient mesoscopic entities^3–5^, even though they are intrinsically diamagnetic^6–16^. Examples include the magneto-induced alignment of liquid-crystalline (LC) materials^3,6–12^, polymers^13,14^ and organic/inorganic objects with a high aspect ratio^4,5,13^. The magneto-induced deformation of a spherical assembly of an amphiphile has also been reported^17^. However, the energy of magnetic fields has been considered too small to be able to influence molecular assembly processes under thermodynamic control. Accordingly, the resulting structures at the molecular level are generally independent of the presence or absence of a magnetic field, albeit only two exceptions have been reported for the occurrence of polymorphs during the solidification of organic molecules in magnetic fields^18,19^.

Here, we show an unprecedented phenomenon that a strong magnetic field can change the thermodynamically determined phase diagrams of diamagnetic liquid crystals. The liquid crystals consist of a discotic triphenylene core and six imidazolium bromide-terminated paraffinic side chains. Upon complexation with diamagnetic LaBr_3_, the resultant triphenylene-based ionic LC assemblies in the absence of a magnetic field exhibit a cubic, an orthorhombic or a hexagonal columnar mesophase, depending on the content of LaBr_3_ and temperature. When the LC composite with a certain content of LaBr_3_ was thermally processed under a strong magnetic field, the original phase diagram was dramatically changed, where the orthorhombic phase was fully replaced by a cubic phase. Through the investigation of the magneto-responsive phase behaviour of the diamagnetic liquid crystals, we demonstrate that the phase diagrams of diamagnetic molecular assemblies can be reprogrammed by applying a magnetic field.

## Results

### Design of the liquid crystals and initial observations of the magneto-responsive phase behaviour

**Fig. 1:**
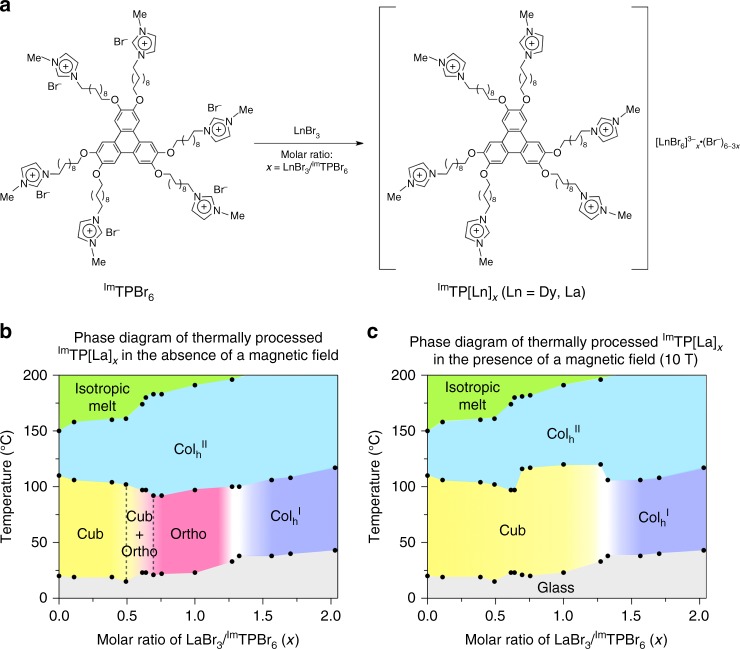
Chemical structures of ^Im^TPBr_6_ and ^Im^TP[Ln]_*x*_ (Ln = Dy, La), and phase diagrams of ^Im^TP[La]_*x*_. **a** Preparation of composites ^Im^TP[Ln]_*x*_. **b**, **c** Phase diagrams of ^Im^TP[La]_*x*_ after thermal processing in the absence (**b**) or presence (**c**) of a 10-T magnetic field. Cub cubic, Ortho orthorhombic, Col_h_ hexagonal columnar. The boundaries of the white regions could not be clearly determined

Previously, we reported that paraffinic hexaalkoxytriphenylene derivatives carrying imidazolium pendants with BF_4_^−^ or PF_6_^−^ counter anions (Supplementary Fig. 1) form LC assemblies that exhibit *Ia*3̄*d* cubic and *P*6*mm* hexagonal columnar phases at lower and higher temperature, respectively^20,21^. Prior to these reports, only a few discotic molecules capable of forming *Ia*3̄*d* cubic LC structures had been known^22–24^. We envisioned that such ionic LC assemblies^25–28^ might become magneto-responsive if the counter anions were replaced with a paramagnetic metal ion. In particular, the hexagonal columnar assembly might be efficiently aligned in a magnetic field, although discotic columns are generally reluctant to align in external electric and magnetic fields^9,10,29,30^. With this expectation, we treated an imidazolium bromide-appended paraffinic triphenylene, ^Im^TPBr_6_ (Fig. 1a), with paramagnetic DyBr_3_ at different molar ratios, *x* = DyBr_3_/^Im^TPBr_6_, which furnished complex anion [DyBr_6_]^3−^ quantitatively, to give [DyBr_6_]^3−^_*x*_•(Br^−^)_6−3*x*_ (*x* ≤ 2.0) (Fig. 1a and see also Supplementary Note 3). Depending on the molar ratio (*x*) and the temperature, the obtained ^Im^TP[Dy]_*x*_ (Fig. 1a) showed a mesophase with an *Ia*3̄*d* cubic (Cub, 0.0 < *x* ≤ 0.5), a *Pbcm* orthorhombic (Ortho, 0.7 ≤ *x* ≤ 0.8) or a *P*6*mm* hexagonal columnar (Col_h_, *x* > 1.0) structure, resulting in the phase diagram shown in Supplementary Fig. 2a (see also Supplementary Figs. 3–6). At 0.5 < *x* < 0.7, ^Im^TP[Dy]_*x*_ displayed complex phase behaviour, in which either the Cub phase or the Ortho phase appeared stochastically or multiple domains of the two mesophases occurred simultaneously (Supplementary Fig. 7). During the magnetic alignment experiments, we serendipitously found an intriguing phenomenon: when ^Im^TP[Dy]_0.75_ was heated once to 180 °C and subsequently allowed to cool to 25 °C (cooling rate ≤ 0.5 °C/min) in a 10-tesla (T) magnet, the Ortho phase disappeared and a Cub phase emerged (Supplementary Figs. 2b, 8).

Initially, we thought that this unexpected phenomenon should arise from the paramagnetic nature of ^Im^TP[Dy]_*x*_. However, we later noticed that even the diamagnetic analogue ^Im^TP[La]_*x*_ (*x* = LaBr_3_/^Im^TPBr_6_; Fig. 1a), which exhibits a more well-defined phase diagram than ^Im^TP[Dy]_*x*_ (see Supplementary Discussion 1), displays a similar magneto-responsive phase behaviour, as shown in Fig. 1b, c. In brief, when ^Im^TP[La]_*x*_ was thermally processed in strong magnetic fields, an Ortho phase, which was originally observed in the phase diagram of ^Im^TP[La]_*x*_ at 0.7 ≤ *x* < 1.3 (Fig. 1b), was completely replaced by a Cub phase (Fig. 1c).

### Phase diagrams of ^Im^TP[La]_*x*_ in the absence and presence of a magnetic field

**Fig. 2:**
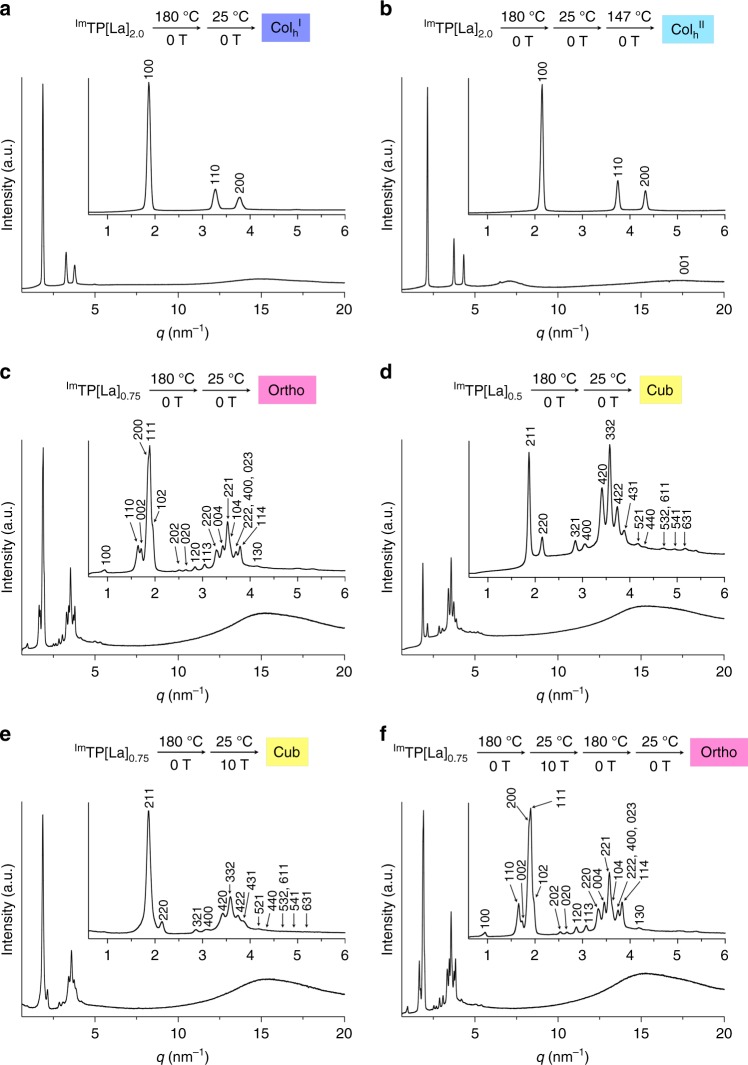
Powder X-ray diffraction (PXRD) patterns of bulk samples of ^Im^TP[La]_*x*_. **a**–**f** PXRD patterns and magnifications of the small-angle region (scattering vector *q* = 0.6–6 nm^−1^) of ^Im^TP[La]_2.0_ at 27 °C on first heating (**a**), ^Im^TP[La]_2.0_ at 147 °C on first heating (**b**), ^Im^TP[La]_0.75_ at 27 °C on first heating (**c**), ^Im^TP[La]_0.5_ at 27 °C on first heating (**d**), ^Im^TP[La]_0.75_ at 27 °C on first heating after thermal processing in a 10-T magnetic field (**e**) and ^Im^TP[La]_0.75_ at 27 °C after thermal processing in a 10-T magnetic field, followed by thermal processing in the absence of a magnetic field (**f**). Prior to the measurements, the samples in (**a**–**d**) were heated once to 180 °C in the absence of a magnetic field. The samples in (**e**, **f**) were heated once to 180 °C in the absence of a magnetic field, and subsequently cooled to 25 °C (cooling rate ≤ 0.5 °C/min) in a 10-T magnetic field. Cub cubic, Ortho orthorhombic, Col_h_ hexagonal columnar, a.u. arbitrary unit. Indices of the reflections are shown in the magnifications of the PXRD patterns

Composites ^Im^TP[La]_*x*_ (0.0 < *x* ≤ 2.0) were prepared by mixing ^Im^TPBr_6_ with appropriate amounts of LaBr_3_ in dehydrated methanol, followed by removal of the solvent under reduced pressure (see Supplementary Note 3). Based on analyses using synchrotron powder X-ray diffraction (PXRD), differential scanning calorimetry (DSC) and polarized optical microscopy (POM), we established the phase diagram of ^Im^TP[La]_*x*_ as shown in Fig. 1b. At *x* = 2.0, ^Im^TP[La]_2.0_ exhibited two different Col_h_ phases at 43–117 °C (Col_h_^I^) and 117–222 °C (Col_h_^II^) (Supplementary Fig. 9). Judging from the absence and presence of a broad diffraction peak arising from π-stacked triphenylene molecules, the Col_h_^I^ and Col_h_^II^ phases feature disordered and ordered intracolumnar stacking, respectively (Fig. 2a, b). When the molar ratio was decreased (0.7 ≤ *x* < 1.3), the Col_h_^I^ phase disappeared and the Ortho phase emerged in the temperature region below the Col_h_^II^ phase (Fig. 2c and Supplementary Figs. 10 and 11). At 0.0 < *x* ≤ 0.5, ^Im^TP[La]_*x*_ exhibited the Cub phase in the temperature region below the Col_h_^II^ phase (Fig. 2d and Supplementary Fig. 12). The phase behaviour of ^Im^TPBr_6_, i.e. *x* = 0.0 for ^Im^TP[La]_*x*_ (Supplementary Fig. 16), was essentially identical to that of ^Im^TP[La]_*x*_ (0.0 < *x* ≤ 0.5). At 0.5 < *x* < 0.7, either the Ortho or the Cub phase appeared stochastically. Most likely, the Ortho phase is not a kinetically formed metastable phase but rather a thermodynamically stable phase, since there was no change in the POM images of the Ortho phase of, e.g. ^Im^TP[La]_0.75_, even after it was allowed to stand at 90 °C (just below the Ortho-to-Col_h_^II^ phase-transition temperature) for 12 h or at 25 °C for 2 years.

As shown in Fig. 1c, a magnetic field changed the phase diagram of diamagnetic ^Im^TP[La]_*x*_ (0.7 ≤ *x* < 1.3). A glass tube (diameter: 4 mm) containing a bulk sample of ^Im^TP[La]_0.75_ (5 mg), for example, was attached to the copper holder of a cryostat with Kapton tape (see Methods and Supplementary Fig. 17a). After the cryostat chamber was evacuated, the cryostat was placed at the centre of the bore (diameter: 10 cm) of a 10-T superconducting magnet in the absence of a magnetic field. Subsequently, the sample was heated to 180 °C, which is just below the clearing point of ^Im^TP[La]_0.75_ (182 °C), and annealed at this temperature for 10 min. Next, a magnetic field of 10 T was applied at the same temperature while the sample was cooled slowly to 25 °C (cooling rate ≤ 0.5 °C/min). The resulting sample at 27 °C showed a PXRD pattern (Fig. 2e) that was substantially different from that observed for the original Ortho phase (Fig. 2c), but essentially identical to that of the Cub phase of ^Im^TP[La]_*x*_ (0.0 < *x* ≤ 0.5) at 27 °C (Fig. 2d). The same result was obtained when ^Im^TP[La]_0.75_ was subjected to sequential heating (180 °C) and cooling (25 °C) in the 10-T magnetic field (Supplementary Fig. 13). When the thermally processed ^Im^TP[La]_0.75_ in the presence of a 10-T magnetic field was once heated to its isotropic melt temperature and subsequently cooled in the absence of a magnetic field, the original phase sequence of ^Im^TP[La]_0.75_ (Supplementary Fig. 10) was observed again (Supplementary Fig. 14). Importantly, when a sample of ^Im^TP[La]_*x*_ (0.0 < *x* ≤ 0.5, Cub or *x* > 1.3, Col_h_^I^) was thermally processed in the presence of a 10-T magnetic field under conditions identical to those for ^Im^TP[La]_0.75_, the original phase remained unchanged (Fig. 1b, c).

### In situ observations of the phase change of ^Im^TP[La]_0.75_ under a magnetic field

**Fig. 3:**
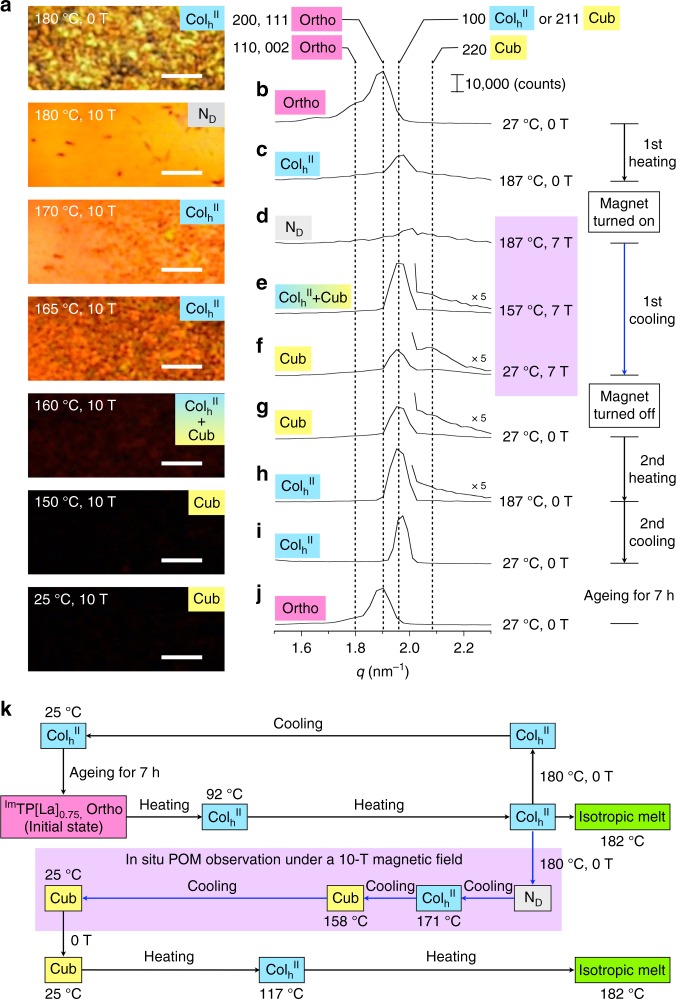
In situ observations of the phase behaviour of ^Im^TP[La]_0.75_ under a strong magnetic field. **a** POM micrographs of a film of ^Im^TP[La]_0.75_ on a glass substrate observed at 180 °C (first heating; heating rate: 20 °C/min) in the absence of a magnetic field, and observed at 180, 170, 165, 160, 150 and 25 °C upon cooling (cooling rate ≤ 0.5 °C/min) in a 10-T magnetic field. Scale bars, 200 µm. **b**–**j** X-ray diffraction patterns of the small-angle region (scattering vector *q* = 1.5–2.3 nm^−1^) of a bulk sample of ^Im^TP[La]_0.75_ in a glass capillary (diameter: 2.5 mm) measured at 27 °C (**b**) and 187 °C (**c**) upon first heating (heating rate: 20 °C/min) in the absence of a magnetic field, measured at 187 °C (**d**), 157 °C (**e**) and 27 °C (**f**) upon first cooling from 187 °C (cooling rate: 0.5 °C/min) in a 7-T magnetic field, measured at 27 °C (**g**) and 187 °C (**h**) upon second heating (heating rate: 20 °C/min) and measured at 27 °C (**i**, **j**) upon second cooling (cooling rate: 0.5 °C/min) in the absence of a magnetic field. In (**j**), the measurement was conducted after ageing for 7 h at 27 °C. Ortho orthorhombic, Col_h_ hexagonal columnar, N_D_ discotic nematic, Cub cubic, a.u. arbitrary unit. Indices of the reflections are shown at the top of the X-ray diffraction peaks. **k** Schematic representation of the phase-transition behaviour of ^Im^TP[La]_0.75_ upon thermal processing in the absence or presence of a 10-T magnetic field. The phase-transition temperatures were determined based on POM observations in the absence or presence of the magnetic field

This magneto-assisted phase-selection was directly monitored by in situ POM in the 10-T magnet. A film sample of ^Im^TP[La]_0.75_ (thickness: ca. 50 µm) on a glass substrate was placed at the centre of the bore (diameter: 10 cm) in the 10-T magnet in such a way that the substrate surface was oriented perpendicular to the magnetic flux (see Methods and Supplementary Fig. 17b). When the film was once heated to ~180 °C, a birefringent texture of the Col_h_^II^ phase of ^Im^TP[La]_0.75_ was observed (Fig. 3a, top). When a 10-T magnetic field was applied at the same temperature, the birefringent texture gradually disappeared, and a uniform orange-coloured POM image appeared (Fig. 3a). These observations indicate that the molecules are homogeneously aligned over the entire film. We presume that this change is due to a phase transition from the Col_h_^II^ to a discotic nematic (N_D_) phase (see Supplementary Discussion 2), which is absent in the phase diagrams of ^Im^TP[La]_*x*_ regardless of the presence or absence of a magnetic field during thermal processing (Fig. 1b, c). When the film sample was cooled in the 10-T magnet, a birefringent texture reappeared at ~170 °C, persisted to ~160 °C, and then disappeared at 158 °C, giving rise to a dark POM image over the entire film down to 25 °C (Fig. 3a). Obviously, the assembling structures of ^Im^TP[La]_0.75_ in the Col_h_^II^ and Ortho phases are optically anisotropic and display birefringent textures in POM, whereas that in the Cub phase is inherently optically isotropic to afford a dark POM image. Thus, the in situ POM observations in the presence of a 10-T magnetic field suggest that the Cub phase originates from the Col_h_^II^ or the Ortho phase at ~160 °C (see Supplementary Discussion 3).

We also monitored the change in the diagnostic X-ray diffraction peaks of ^Im^TP[La]_0.75_ at *q* = 1.5–2.3 nm^−1^ during thermal processing in an applied magnetic field of 7 T. For this in situ X-ray diffraction measurement, we designed a dedicated experimental setup by modifying an 8-T magnet (bore diameter: 2.5 cm) and a cryostat (see Methods and Supplementary Fig. 17c). At 27 °C, prior to application of the 7-T magnetic field, ^Im^TP[La]_0.75_ showed broad peaks at *q* = 1.7–2.0 nm^−1^ (Fig. 3b), which were assigned to reflections 110, 002, 200 and 111 of the *Pbcm* orthorhombic structure. Upon heating to 187 °C in the absence of a magnetic field, these diffraction peaks disappeared, and a new broad peak at *q* = 1.96 nm^−1^ emerged due to the Ortho → Col_h_^II^ phase transition (Fig. 3c). The intensity of this diffraction peak became negligible when the sample was allowed to stand for 10 min at 187 °C after the application of a 7-T magnetic field (Fig. 3d). This observation indicates that the material loses its two-dimensional (2D) hexagonal structural order, which is consistent with the formation of the N_D_ phase observed by in situ POM. When the sample was subsequently cooled to 157 °C in the presence of the magnetic field, a strong peak at *q* = 1.96 nm^−1^ reappeared, together with a weak diffraction at *q* = 2.1 nm^−1^ (Fig. 3e). Although the reciprocal *q*-spacing ratio of these two peaks (√6:√8) agrees well with that expected for reflections 211 and 220 of the *Ia*3̄*d* cubic structure, their intensity ratio is different from that observed for the magnetically induced Cub phase of ^Im^TP[La]_0.75_ (Fig. 2e). The observed X-ray diffraction peaks can be explained by considering the coexistence of the Col_h_^II^ and Cub phases at 157 °C in the presence of the magnetic field. Importantly, upon further cooling to 27 °C, the intensity of the stronger peak decreased, whereas that of the weaker peak increased, resulting in an intensity ratio of 5.0 at 27 °C (Fig. 3f), which is virtually identical to that observed for the magnetically induced Cub phase of ^Im^TP[La]_0.75_ (4.8; Fig. 2e). Therefore, in the 7 T magnetic field, the Cub phase of ^Im^TP[La]_0.75_ starts to emerge from the Col_h_^II^ phase at ~160 °C and develops over the entire material at 27 °C. The diffraction peaks of the Cub phase persisted stably at 27 °C even after the magnetic field intensity was decreased to 0.0 T (Fig. 3g). However, in a second heating and cooling cycle in the absence of a magnetic field, the system recovered the diffractions that were characteristic of the original Ortho phase (Fig. 3h–j). Meanwhile, we confirmed that, upon second heating in the presence of the 7-T magnetic field, the magnetically induced Cub phase of ^Im^TP[La]_0.75_ is maintained up to at least 157 °C (Supplementary Fig. 18), which is higher than the Cub-to-Col_h_^II^ phase-transition temperature of ^Im^TP[La]_*x*_ (0.0 < *x* ≤ 0.5) (Fig. 1b) as well as that of the magnetically induced Cub phase of ^Im^TP[La]_0.75_, observed upon heating in the absence of a magnetic field. The fact that the temperature range of the Cub phase expands in the presence of the magnetic field suggests this phase is substantially stabilized under the conditions employed.

### Magneto-responsive phase behaviour of ^Im^TP[La]_0.75_

Based on an experiment using a bulk sample of ^Im^TP[La]_0.75_ in a glass capillary (diameter: 0.7 mm), the magnetically induced Cub phase of ^Im^TP[La]_0.75_ did not spontaneously transform back into the Ortho phase even after standing at 25 °C for more than 3 months outside the magnet, while it changed after heating to 180 °C and subsequent cooling to 25 °C in the absence of a magnetic field, resulting in a PXRD pattern (Fig. 2f), which is identical to that of the original Ortho phase of ^Im^TP[La]_0.75_ (Fig. 2c). These results obtained by in situ POM and XRD experiments using a film and a bulk sample of ^Im^TP[La]_0.75_, respectively, clearly indicate that the structural information of the LC material, memorized under the application of a magnetic field, can be initialized by thermal processing without a magnetic field.

With the above results in mind, we can now provide a full picture of the magneto-responsive phase behaviour of ^Im^TP[La]_0.75_, as illustrated in Fig. 3k. Notably, the magneto-assisted phase-selection depends critically on the strength of the applied magnetic field as well as on the annealing temperature. For instance, upon thermal processing with a 5-T magnetic field, the Cub phase of ^Im^TP[La]_0.75_ was stochastically selected over the Ortho phase and, in some cases, an LC assembly composed of a mixture of domains of the Ortho and Cub phases was obtained (Supplementary Fig. 15a). In a magnetic field stronger than 5 T, only the Cub phase emerged. Accordingly, the magneto-assisted phase-selection of ^Im^TP[La]_0.75_ might require magnetic fields stronger than 5 T. Meanwhile, when ^Im^TP[La]_0.75_ was annealed for 10 min at 150 °C in a 10-T magnetic field, a mixture of domains of the Ortho and Cub phases formed (Supplementary Fig. 15b). Upon annealing for 10 min at a lower temperature (e.g. 90 °C), the original Ortho phase of ^Im^TP[La]_0.75_ was maintained even after thermal processing in a 10-T magnetic field (Supplementary Fig. 15c).

### Superconducting quantum interference device (SQUID) measurements of ^Im^TP[La]_*x*_

**Fig. 4:**
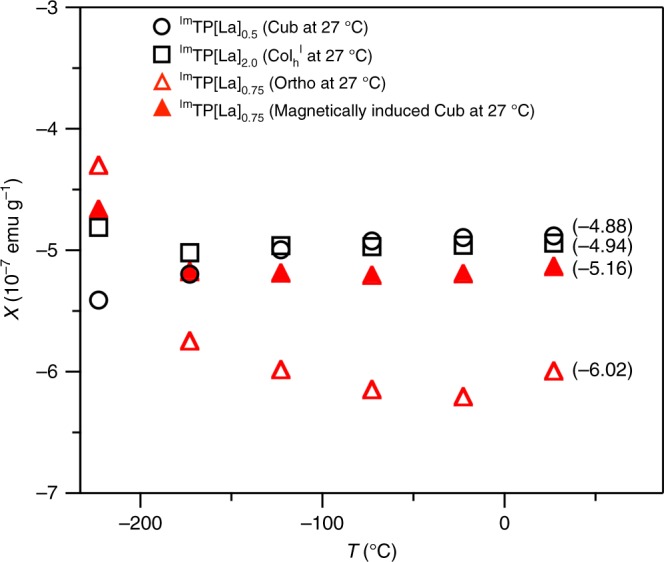
SQUID measurements of ^Im^TP[La]_*x*_. *χ*−*T* curves for ^Im^TP[La]_*x*_ after thermal processing in the absence of a magnetic field (*x* = 0.5: open circle, *x* = 2.0: open square and *x* = 0.75: open triangle) and for ^Im^TP[La]_0.75_ after thermal processing in a 10-T magnetic field (filled triangle). Values in parentheses represent the *χ* values (10^−7^ emu/g) of the samples at 27 °C

To gain insight into this magneto-assisted phase-selection, we evaluated the effect of a magnetic field on the free energy (*E*_mag_) of ^Im^TP[La]_*x*_ using the equation *E*_mag_ = −*χB*^2^⁄2*μ*_0_, where *χ* is the mass magnetic susceptibility, *B* is the magnetic flux density and the physical constant *μ*_0_ is the magnetic permeability of vacuum^15,31^. Using a SQUID (see Methods), we measured *χ* values at various temperatures for ^Im^TP[La]_*x*_ (*x* = 0.5, 0.75 and 2.0) after thermal processing in the absence of a magnetic field, as well as that of ^Im^TP[La]_0.75_ after thermal processing in a 10-T magnetic field (Fig. 4). We confirmed that no macroscopic alignment of the anisotropic Ortho phase occurs at the measurement temperature range even under the application of a 5-T magnetic field, and thus the SQUID profiles reflect the intrinsic magnetic properties of the materials. Due to the diamagnetic nature of ^Im^TP[La]_*x*_, the *χ* value for each sample was negative at all temperatures examined. The diamagnetic signals, |*χ*|, of ^Im^TP[La]_0.5_ and ^Im^TP[La]_2.0_, which adopt Cub and Col_h_^I^ structures at 27 °C, respectively, were comparable to each other. The |*χ*| values of ^Im^TP[La]_0.75_, which originally adopts the Ortho structure at 27 °C, were slightly greater than those of ^Im^TP[La]_0.5_ and ^Im^TP[La]_2.0_. The *χ* values of ^Im^TP[La]_0.5_, ^Im^TP[La]_2.0_ and ^Im^TP[La]_0.75_ at 27 °C were determined to be −4.88 × 10^−7^, −4.94 × 10^−7^ and −6.02 × 10^−7^ emu/g, respectively. Since the *B*^2^⁄2*μ*_0_ values are identical under the measurement conditions, the Ortho phase of ^Im^TP[La]_0.75_ should be more destabilized than the other LC phases in terms of *E*_mag_. Remarkably, when ^Im^TP[La]_0.75_ was heated to 180 °C and subsequently cooled to 25 °C in the presence of a 10-T magnetic field, the resulting material showed a *χ*–*T* curve similar to that observed for the Cub phase of ^Im^TP[La]_0.5_ (Fig. 4). Furthermore, the |*χ*| value of ^Im^TP[La]_0.75_ at 27 °C was decreased from 6.02 × 10^−7^ to 5.16 × 10^−7^ emu/g after thermal processing in the presence of a 10-T magnetic field. The change in the *χ*–*T* curve for ^Im^TP[La]_0.75_ before and after thermal processing in the magnetic field strongly supports the occurrence of magneto-assisted phase-selection, where the initial Ortho phase is reprogrammed into the Cub phase (Fig. 3k).

## Discussion

Based on an analogy to conventional thermotropic and lyotropic liquid crystals that exhibit a phase transition in response to thermal stimuli and the presence of solvents, respectively, the unprecedented phase behaviour of the present LC material can be referred to as ‘magnetotropic’. The discovery that a magnetic field can change the phase behaviour of a diamagnetic molecular assembly should not only impact various scientific areas such as chemistry, condensed matter physics and materials science, but also substantially expand the understanding of the interplay between soft matter and magnetic fields, which could ultimately provide a concept for the design of organic materials.

## Methods

### Thermal processing of ^Im^TP[Ln]_*x*_ in the presence of a 10-T magnetic field

A glass tube (diameter: 4 mm), containing a bulk sample of ^Im^TP[Ln]_*x*_ (5 mg), was attached to the copper sample holder of a cryostat chamber (ST-300; JANIS Research Co., Inc.) with Kapton tape (Supplementary Fig. 17a). The cryostat chamber was subsequently evacuated by a turbo-molecular pump to maintain a pressure of ~10^−4^ mbar, before being placed at the centre of the bore (diameter: 10 cm) of a 10-T superconducting magnet (JMTD-10T100RK; JASTEC, Inc.) in such a way that the long axis of the glass tube containing the sample was oriented parallel to the magnetic flux. The temperature of the sample holder was controlled with an external temperature-control unit (1900-7; Scientific Instruments, Inc.). Unless otherwise stated, the sample was heated once to 180 °C in the absence of a magnetic field, before being cooled to 25 °C (cooling rate ≤ 0.5 °C/min) in the presence of a 10-T magnetic field. After thermal processing, the sample was collected and stored under argon in a sealed vial at 25 °C.

### Synchrotron radiation powder X-ray diffraction (PXRD) experiments of bulk samples of ^Im^TPBr_6_ and ^Im^TP[Ln]_*x*_ (Ln = Dy or La)

Variable-temperature one-dimensional (1D) X-ray diffraction patterns were measured using beamline 44B2 in SPring-8 (Hyogo, Japan) equipped with an imaging-plate area detector^32^. The wavelength (1.08 Å) of incident X-rays was calibrated using cerium oxide (standard reference material 674b). The sample-to-detector distance was 286.5(1) mm. Unless otherwise stated, bulk samples in a glass capillary (diameter: 0.7 mm) were measured while spinning at a rate of 60 rpm.

### Structural characterization of the Ortho phases of ^Im^TP[Dy]_0.75_ and ^Im^TP[La]_0.75_

A shear-oriented film sample of ^Im^TP[Dy]_0.75_ or ^Im^TP[La]_0.75_ was prepared by applying a shear stress to a bulk material of ^Im^TP[Dy]_0.75_ or ^Im^TP[La]_0.75_ at 150 °C on a sapphire substrate, and the resulting film was slowly cooled to 25 °C and aged at 25 °C for several days. A 2D X-ray diffraction image of the film (Supplementary Fig. 5 for ^Im^TP[Dy]_0.75_ or Supplementary Fig. 11 for ^Im^TP[La]_0.75_) was obtained using beamline 45XU in SPring-8 (Hyogo, Japan) equipped with an R-AXIS IV++ (Rigaku) imaging-plate area detector. The scattering vector, *q* = 4*π*sin*θ*/*λ*, and the position of the incident X-ray beam on the detector were calibrated using several orders of layer reflections from silver behenate (*d* = 58.380 Å), where 2*θ* and *λ* refer to the scattering angle and wavelength of the X-ray beam (1.0 Å), respectively. The sample-to-detector distance was 0.5 m. The cell parameters were refined using CellCalc ver. 2.10 software^33^.

### In situ polarized optical microscopic (POM) observation in the presence of a 10-T magnetic field

To visualize the phase-transition events of ^Im^TP[La]_0.75_ inside a 10-T magnet (JMTD-10T100RK; JASTEC, Inc.), we designed a dedicated heater unit (Supplementary Fig. 17b, left) consisting of a heater block, polariser and borescope (TechnoPack X; Karl Storz Co.). A film of ^Im^TP[La]_0.75_ on a 100 μm-thick glass substrate was attached to the heater block (Supplementary Fig. 17b, left). The heater was then placed inside the bore of the magnet (Supplementary Fig. 17b, right) in such a way that the surface of the substrate was oriented perpendicular to the magnetic flux, while the film sample was located at the centre of the bore. Due to the limitations of the experimental setup used, the exact temperature could not be ascertained.

### In situ X-ray diffraction measurement in the presence of a 7-T magnetic field

In situ variable-temperature X-ray diffraction experiments in the presence of a magnetic field were carried out using beamline 19LXU in SPring-8 (Hyogo, Japan)^34^. An Oxford cryostat (Spectrostat-HT500V) and a rod-type sample holder were specifically modified and prepared, respectively, so as to adapt to the high-temperature measurements in a superconducting magnet. A bulk sample of ^Im^TP[La]_0.75_ in a glass capillary (diameter: 2.5 mm) was placed inside a copper tube (Supplementary Fig. 17c, left). The tube was then attached to the rod-type sample holder (Supplementary Fig. 17c, centre) and inserted into the modified cryostat (Supplementary Fig. 17c, right). The cryostat containing the sample was subsequently placed inside a 25-mm variable-temperature insert (VTI) sample space of an 8-T superconducting magnet (PN5090; Oxford Instruments), containing windows of multilayer films of beryllium and Kapton. The superconducting magnet was then mounted on a HUBER crystallography diffractometer equipped with an avalanche photodiode (APD) detector (Supplementary Fig. 17c, right). The VTI sample space (~1 mbar) and the cryostat chamber were evacuated (~10^−5^ mbar), and the temperature of the sample was controlled by an external temperature-control unit (Model 335 Cryogenic Temperature Controller; Lake Shore Cryotronics, Inc.). Due to the limitations of the experimental setup used, the exact temperature could not be ascertained. The sample was exposed to the X-ray beam (Si double-crystal monochromated X-rays: *λ* = 1.0 Å) through the windows of the magnet, VTI sample space and cryostat. The X-ray diffraction patterns were collected using the APD detector (sample-to-detector distance: 1.75 m).

### Superconducting quantum interference device (SQUID) measurements

The mass magnetic susceptibility *χ*(*T*) of ^Im^TP[La]_*x*_ as a function of temperature *T* was measured using a Quantum Design MPMS-5S SQUID magnetometer with a maximum field of 5 T. The *χ–T* curve of each ^Im^TP[La]_*x*_ sample was measured upon cooling (cooling rate: 0.5 °C/min) from 27 to −223 °C in the presence of a magnetic field (*B* = 0.5 T). Prior to each measurement, bulk samples (5–13 mg) of ^Im^TP[La]_*x*_ were thermally processed at 180 °C either in the presence or absence of a 10-T magnetic field. Each sample was packed in a glass capillary (ca. 8 mm in height, ca. 3 mm in outside diameter and ca. 30 mg in weight) using a non-metallic toothpick to avoid magnetic contamination from the outside. The glass capillary containing the sample was sandwiched between double straws made of Kapton film (ca. 180 mm in length and 0.1 mm in thickness) and fixed in the centre of the straws. For all measurements, the Reciprocating Sample Option (RSO) with 4-cm sample movement and 1 Hz repetition frequency were used to achieve the high sensitivity of ~10^−8^ emu. The magnetization of the glass capillary alone was measured first, then the magnetization of the sample was measured in the glass capillary, and the magnetization of only the sample was obtained from the difference between them.

## Electronic supplementary material

Supplementary Information

## Data Availability

All relevant data are included in full within this paper and in the Supplementary Information.
