# Supplementary material for: Rewriting the phase diagram of a diamagnetic liquid crystal by a magnetic field
Source: Nat Commun. 2018 Oct 25;9:4431. doi: 10.1038/s41467-018-06976-7 (PMC6202376; doi:10.1038/s41467-018-06976-7)
Supplement: Supplementary file 1 — Supplementary Information [file 41467_2018_6976_MOESM1_ESM.pdf]

Supplementary Information

Rewriting the phase diagram of a diamagnetic liquid crystal by a magnetic field

Fatin Hajjaj, Takashi Kajitani, Hiroyuki Ohsumi, Yoshikazu Tanaka, Kenichi Kato, Masaki Takata, Hideaki Kitazawa, Taka-hisa Arima, Takuzo Aida* & Takanori Fukushima*

e-mail: fukushima@res.titech.ac.jp (T.F.); aida@macro.t.u-tokyo.ac.jp (T.Aida)

Supplementary Note 1: Materials. $DyBr_3$ (anhydrous, 99.99%) and $LaBr_3$ (anhydrous, 99.99%) were purchased from Aldrich Chemical and used as received. Dehydrated MeOH (99.8%) was obtained from Wako Pure Chemical Industries and used as received. Solid substrates were purchased from Matsunami Glass Ind., Ltd. (glass) and Sigma Koki Co., Ltd. (sapphire) and used as received. Substrates were cut to the designated size prior to use. $^{Im}TPBr_6$ was prepared according to previously reported procedures[1,2]: ^{1}H NMR (500 MHz, CD_3CN): δ (ppm) 9.05 (s, 6H), 7.89 (s, 6H), 7.43 (dd, J = 1.7, 1.7 Hz, 6H), 7.37 (dd, J = 1.7, 1.7 Hz, 6H), 4.25 (t, J = 6.3 Hz, 12H), 4.14 (t, J = 7.4 Hz, 12H), 3.85 (s, 18H), 1.89−1.78 (m, 24H), 1.58−1.27 (m, 72H). ^{13}C NMR (125 MHz, CD_3CN): δ (ppm) 149.50, 136.29, 124.14, 123.72, 122.74, 107.31, 69.67, 50.03, 36.34, 30.11, 29.76, 29.74, 29.64, 29.60, 29.15, 26.44, 26.23. ESI-TOF mass: calcd. for $C_{102}H_{162}Br_6N_{12}O_6$ $[M–Br]^+$: m/z = 2051.86; found: 2051.81. Anal. calcd. for $C_{102}H_{162}Br_6N_{12}O_6 \bullet 4H_2O$: C, 55.59; H, 7.77; N, 7.63. Found: C, 55.41; H, 7.99; N, 7.56. FIR (polyethylene pellet): ν (cm^{-1}) 284.9, 226.2, 91.1. Prior to use, $^{Im}TPBr_6$ was dried for 48 h *in vacuo* (10^{-3} mbar, 70 °C).

Supplementary Note 2: Methods. $^{Im}TP[Ln]_x$ were prepared under argon in a glove box (Unilab, MBraun). Nuclear magnetic resonance (NMR) spectroscopy measurements were carried out on a JEOL JNM-ECA500 spectrometer (500 MHz for ^{1}H and 125 MHz for ^{13}C). Chemical shifts (δ) are expressed relative to the resonances of the residual non-deuterated solvent for ^{1}H [CD_3CN: ^{1}H(δ) = 1.94 ppm] and ^{13}C [CD_3CN: ^{13}C(δ) = 1.4 and 118.7 ppm]. Absolute values of the coupling constants are given in Hertz (Hz), regardless of their sign. Multiplicities are abbreviated as singlet (s), doublet of doublets (dd), triplet (t) and multiplet (m). Electrospray-ionization time-of-flight (ESI-TOF) mass spectrometry measurements were carried out on a JEOL AccuTOF JMS-T100L mass spectrometer. Far-infrared (FIR) spectra were recorded at 25 °C on a JASCO FARIS-1 Fourier-transform infrared spectrometer (range: 2–400 cm^{-1}) equipped with a Si-composite bolometer detector. Elemental analyses were carried out on a Yanaco CHN CORDER MT-6. Differential scanning calorimetry (DSC) measurements were recorded on a Mettler–Toledo DSC 1 differential scanning calorimeter, where temperature and enthalpy were calibrated with In (430 K, 3.3 J/mol) and Zn (692.7 K, 12 J/mol) standard samples in sealed Al pans. Cooling and heating profiles were recorded and analysed using the Mettler–Toledo STAR^e software system. Polarized optical microscopy (POM) was performed on a Nikon Eclipse LV100POL optical polarizing microscope equipped with a Mettler–Toledo FP90 controller attached to a FP82HT hot stage.

Unless otherwise stated, POM measurements were carried out on film samples sandwiched between glass substrates.

Supplementary Note 3: Preparation of ^{Im}TP[Ln]$_x$ (Ln = Dy or La). Typically, $^{Im}TPBr_6$ (23.45 μmol) was mixed with the corresponding amount of $LnBr_3$ (*e.g.*, 11.55, 17.65 or 47.66 μmol for x = 0.5, 0.75 and 2.0, respectively) in dehydrated MeOH (2.5 mL), and the mixture was stirred for 24 h at 25 °C. Subsequently, all volatile materials were removed under reduced pressure, and the residue was dried for 4 h at 70 °C under reduced pressure (10^{-3} mbar), to give ^{Im}TP[Ln]$_x$ (Ln = Dy or La) as viscous solids. FIR spectra of ^{Im}TP[Ln]$_{2.0}$ (Supplementary Figure 19) showed an unsymmetrical stretching vibration (F_{1u}) at 160 and 159 cm^{-1} for ^{Im}TP[Dy]$_{2.0}$ and ^{Im}TP[La]$_{2.0}$, respectively, which arises from octahedral $[LnBr_6]^{3-}$ (refs. 3 and 4). The absence of any vibrational bands associated with $LnBr_3$ indicates a quantitative conversion of $LnBr_3$ into $[LnBr_6]^{3-}$. This held true for ^{Im}TP[Ln]$_x$ (x < 2.0) (Supplementary Figure 19), where $[LnBr_6]^{3-}{}_x$•$(Br^-)_{6-3x}$ were formed as the counter anions of the imidazolium pendants.

Supplementary Note 4: Phase Diagrams. Samples were prepared and measured at least for every 0.2 variation in the molar ratio (x) across the phase diagrams. In the vicinity of the phase boundaries, samples were prepared with 0.1 increments in x. The temperature region of each phase was determined based on the DSC and PXRD data obtained from the first heating of the samples, which were thermally processed up to 180 °C in the absence or presence of a 10-T magnetic field.

Supplementary Discussion 1. To examine magneto-assisted phase-selection in diamagnetic molecular assemblies, we only focused on the magneto-responsive phase behaviour of ^{Im}TP[La]$_x$. Furthermore, ^{Im}TP[Dy]$_x$ with paramagnetic Dy^{3+} ions did not show a well-defined composition-dependency in the phase diagram, where biphasic behaviours were observed over a wide range of values for the molar ratio (x) even in the absence of a magnetic field (Supplementary Figure 2). Nevertheless, for the sake of completion, the phase behaviours of ^{Im}TP[Dy]$_x$ after thermal processing in the presence and absence of a 10-T magnetic field are summarized in Supplementary Figures 2–8.

Supplementary Discussion 2. A magnetic field has been shown to orient diamagnetic aromatic molecules in such a way that their π-planes align parallel to the magnetic flux[5,6]. Based on this fact, and by analogy to the magnetocaloric effect, which is generally known for magnetic materials[7], we propose the following scenario: upon the application of a 10-T magnetic field, the columnar assemblies in the Col_h^{II} phase might align perpendicular to the magnetic flux, causing a loss of entropy, which is accompanied by an increase in the temperature of the system. Possibly due to this thermal effect, the system can undergo a phase transition from the Col_h^{II} phase to the isotropic melt, where the applied magnetic field forces the directors of the molecules to orient. Consequently, the system behaves as a discotic nematic (N_D), which is the least-ordered discotic LC phase, where the directors of the mesogenic cores simply align parallel to each other without any positional correlations between the constituent molecules.

Supplementary Discussion 3. While bulk samples of $^{Im}TP[La]_{0.75}$ as well as thick sandwich films of $^{Im}TP[La]_{0.75}$ with a thickness of ≥25 µm exhibit completely reproducible magneto-induced phase-selection, thin sandwich films of $^{Im}TP[La]_{0.75}$ with a thickness of, *e.g.*, 10 µm, did not show this behaviour. As is often observed in the structurization of liquid crystals on substrates, the structurization of $^{Im}TP[La]_{0.75}$ is likely affected by the substrate surface to cause the thickness-dependence for the film samples.

Supplementary Figures and Tables

Chemical Structures of ^{Im}TP[X] (X = BF_4 or PF_6)

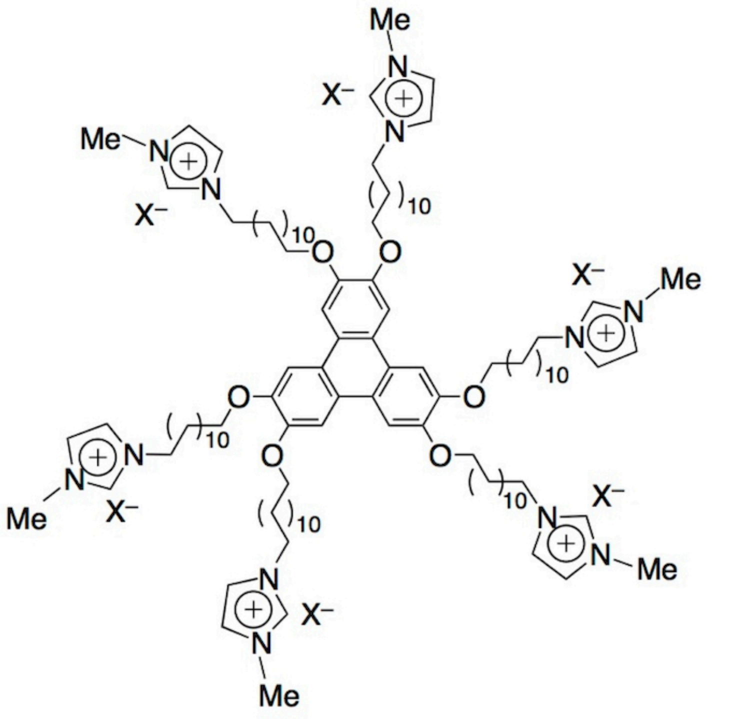

^{Im}TP[BF_4] (X = BF_4)
^{Im}TP[PF_6] (X = PF_6)

Supplementary Figure 1 | Chemical Structures of ^{Im}TP[BF_4] and ^{Im}TP[PF_6].

Phase diagrams of $^{Im}TP[Dy]_x$

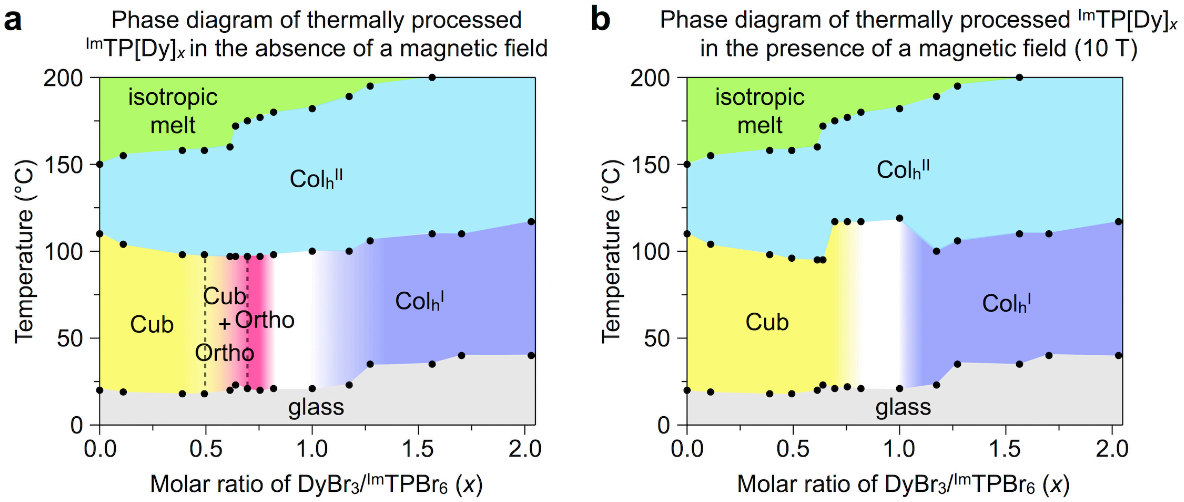

Supplementary Figure 2 | Phase diagrams of $^{Im}TP[Dy]_x$. **a**,**b**, Phase diagrams of $^{Im}TP[Dy]_x$ after thermal processing in the absence (**a**) or presence (**b**) of a 10-T magnetic field. Cub: cubic, Ortho: orthorhombic, Col_h: hexagonal columnar. The boundaries of the white regions could not be clearly determined. For further details, see the Supplementary Figures and Tables for $^{Im}TP[Dy]_x$.

^{Im}TP[Dy]$_{0.5}$

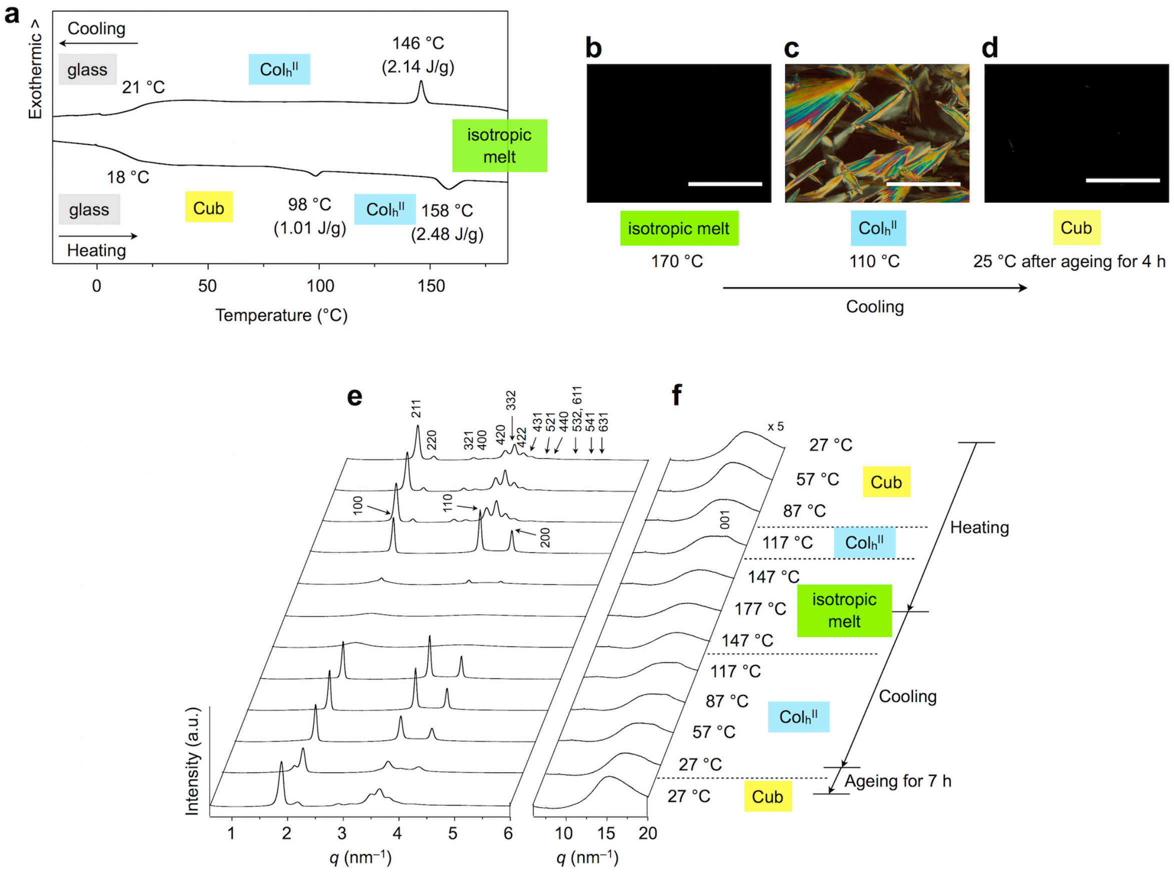

Supplementary Figure 3 | Structural characterization and phase behaviour of thermally processed ^{Im}TP[Dy]$_{0.5}$ in the absence of a magnetic field. **a**, DSC profile (second heating/cooling cycle) of ^{Im}TP[Dy]$_{0.5}$, measured at a scan rate of 10 °C/min; Cub: cubic, Col$_h$: hexagonal columnar. Values in parentheses represent the change in enthalpy (Δ*H*). **b–d**, POM micrographs of ^{Im}TP[Dy]$_{0.5}$ at 170 °C (**b**) and 110 °C (**c**) upon cooling from 180 °C, and at 25 °C after ageing for 4 h (**d**); scale bars, 100 μm. **e**,**f**, Variable-temperature PXRD patterns for the *q* = 0.6–6 nm^{-1} (**e**) and *q* = 6–20 nm^{-1} (**f**) regions of a bulk sample of ^{Im}TP[Dy]$_{0.5}$, measured upon heating and subsequent cooling (heating/cooling rate: 10 °C/min); a.u., arbitrary unit. Indices of the reflections are shown in the PXRD patterns.

Supplementary Table 1 | XRD data for a bulk sample of ^{Im}TP[Dy]$_{0.5}$ upon heating

T (°C)	q (nm^{-1})	$d_{obs.}$ (nm)	$d_{calc.}$ (nm)	hkl
27	1.86	3.39	3.34	211
(Cub)*	2.15	2.92	2.90	220
	2.86	2.20	2.19	321
	3.06	2.05	2.05	400
	3.43	1.83	1.83	420
	3.59	1.75	1.75	332
	3.75	1.68	1.67	422
	3.88	1.62	1.61	431
	4.16	1.51	1.50	521
	4.29	1.47	1.45	440
	4.77	1.32	1.33	532
	4.77	1.32	1.33	611
	5.02	1.25	1.26	541
	5.20	1.21	1.21	631
117	2.10	3.00	2.98	100
(Col$_h^{II}$)†	3.65	1.72	1.72	110
	4.22	1.49	1.49	200
	17.62	0.36	0.36	001

*$Ia\bar{3}d$ cubic cell parameter at 27 °C: a = 8.19 nm; †$P6mm$ hexagonal cell parameters at 117 °C: a = 3.44 nm and c = 0.36 nm.

^{Im}TP[Dy]$_{0.75}$

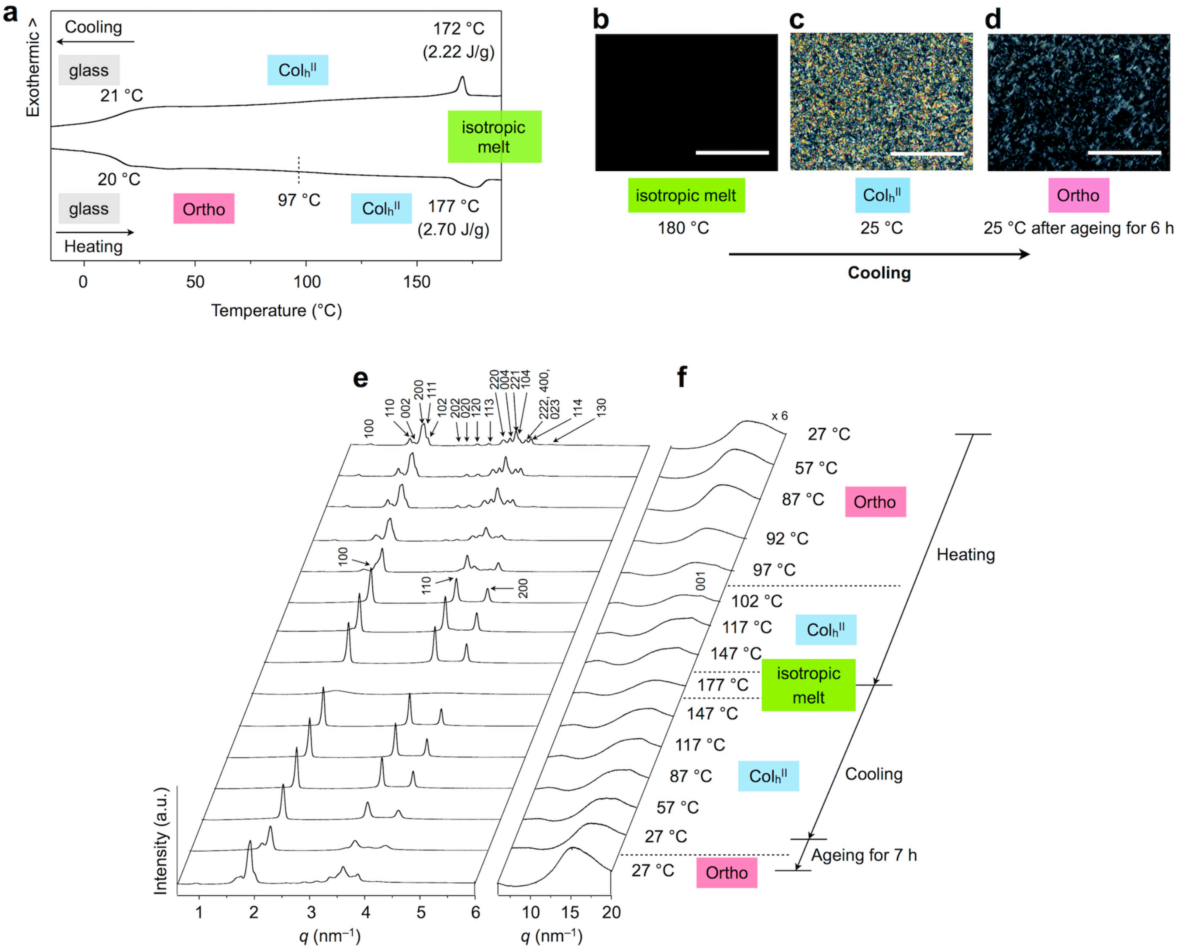

Supplementary Figure 4 | Structural characterization and phase behaviour of thermally processed ^{Im}TP[Dy]$_{0.75}$ in the absence of a magnetic field. **a**, DSC profile (second heating/cooling cycle) of ^{Im}TP[Dy]$_{0.75}$, measured at a scan rate of 10 °C/min; Ortho: orthorhombic, Col_h: hexagonal columnar. Values in parentheses represent the changes in enthalpy (ΔH). The Ortho→Col_h^{II} phase-transition temperature was determined from the PXRD data in **e** and **f**. **b–d**, POM micrographs of ^{Im}TP[Dy]$_{0.75}$ at 180 °C (**b**) and 25 °C (**c**) upon cooling from 185 °C, and at 25 °C after ageing for 6 h (**d**); scale bars, 100 µm. **e**,**f**, Variable-temperature PXRD patterns for the q = 0.6–6 nm^{-1} (**e**) and q = 6–20 nm^{-1} (**f**) regions of a bulk sample of ^{Im}TP[Dy]$_{0.75}$, measured upon heating and subsequent cooling (heating/cooling rate: 10 °C/min); a.u., arbitrary unit. Indices of the reflections are shown in the PXRD patterns.

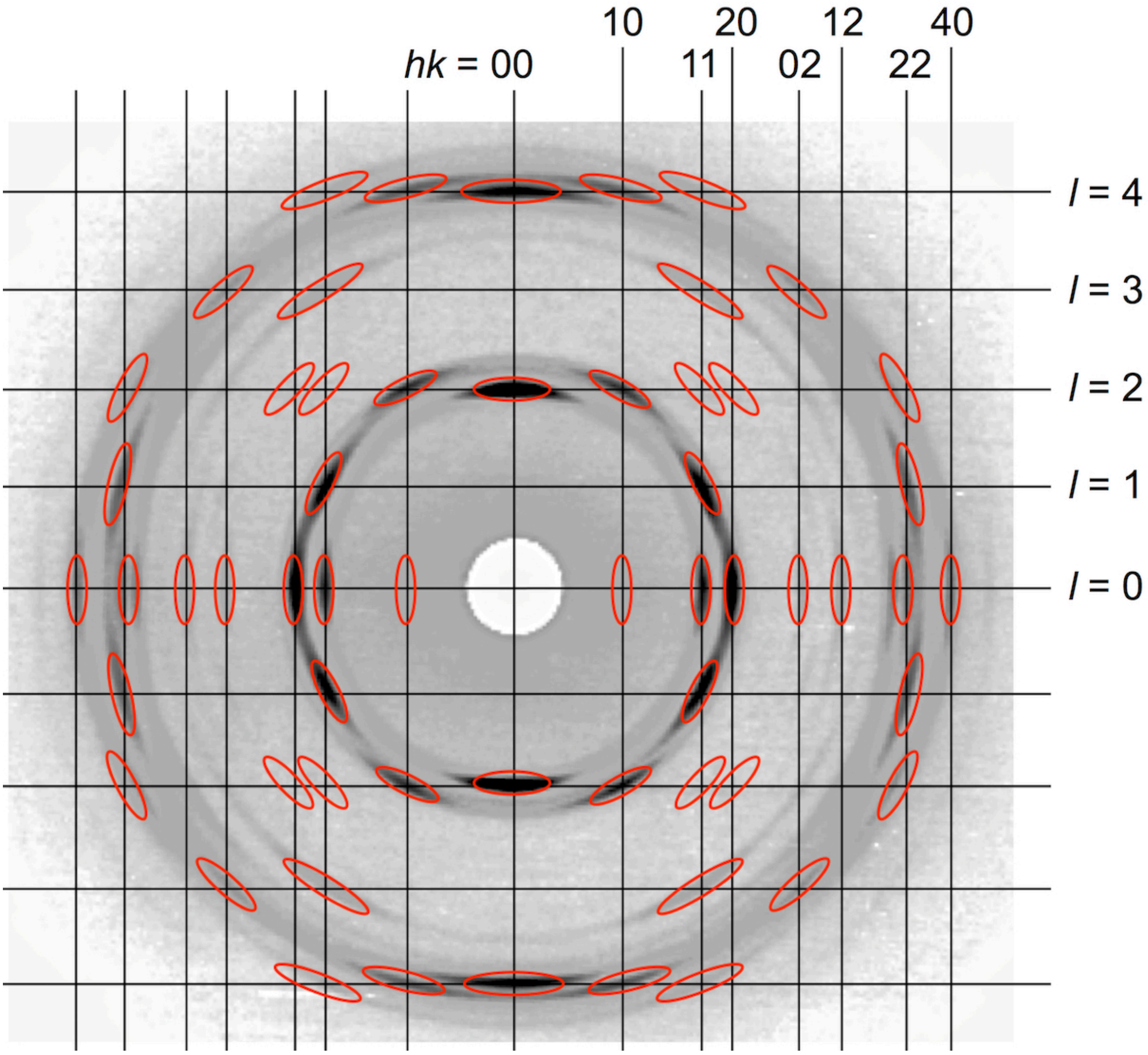

Supplementary Figure 5 | Through-view XRD image observed at 25 °C for a shear-oriented film of the Ortho phase of $^{Im}TP[Dy]_{0.75}$ on a sapphire substrate. *h*, *k* and *l* represent the indices of reflections. The diffraction arcs in an equatorial direction with *d*-spacings of 3.7, 3.3, 1.8 and 1.7 nm were assigned to diffractions from the (110), (200), (220) and (400) planes of a 2D rectangular structure, respectively. The two intense arcs in the meridional direction with *d*-spacings of 3.6 and 1.8 nm were assigned to the diffractions from the (002) and (004) planes of a 1D periodic (layer) structure, respectively, which develops perpendicular to the 2D rectangular lattice. All diffractions observed in the XRD image could be fully indexed by considering a *Pbcm* lattice with parameters *a* = 6.6 nm, *b* = 4.6 nm and *c* = 7.2 nm.

Supplementary Table 2 | XRD data for a bulk sample of $^{Im}TP[Dy]_{0.75}$ upon heating

T (°C)	q (nm^{-1})	$d_{obs.}$ (nm)	$d_{calc.}$ (nm)	hkl
27	0.95	6.60	6.63	100
(Ortho)*	1.66	3.78	3.77	110
	1.73	3.63	3.61	002
	1.89	3.33	3.32	200
	1.92	3.28	3.34	111
	2.03	3.10	3.17	102
	2.54	2.47	2.44	202
	2.69	2.34	2.29	020
	2.88	2.18	2.16	120
	3.09	2.03	2.03	113
	3.36	1.87	1.88	220
	3.48	1.81	1.81	004
	3.58	1.76	1.82	221
	3.63	1.73	1.74	104
	3.76	1.67	1.67	222
	3.76	1.67	1.66	400
	3.76	1.67	1.66	023
	3.85	1.63	1.63	114
	4.21	1.49	1.49	130
147	2.13	2.95	2.95	100
(Col_h^{II})†	3.69	1.70	1.70	110
	4.26	1.48	1.48	200
	17.66	0.36	0.36	001

**Pbcm* orthorhombic cell parameters at 27 °C: a = 6.63 nm, b = 4.59 nm and c = 7.23 nm; †*P6mm* hexagonal cell parameters at 147 °C: a = 3.41 nm and c = 0.36 nm.

^{Im}TP[Dy]$_{2.0}$

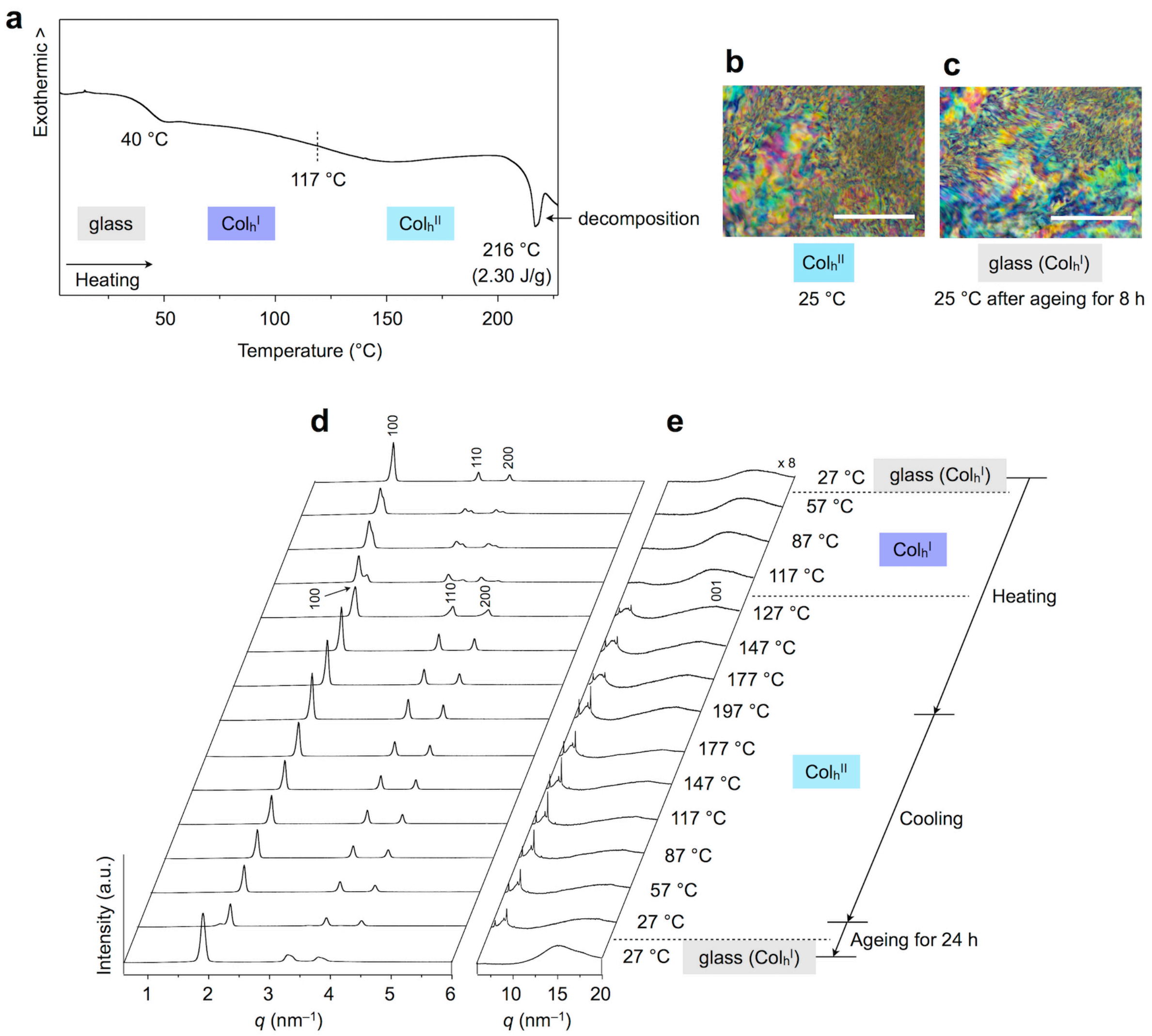

Supplementary Figure 6 | Structural characterization and phase behaviour of thermally processed ^{Im}TP[Dy]$_{2.0}$ in the absence of a magnetic field. **a**, DSC profile (first heating) of ^{Im}TP[Dy]$_{2.0}$, measured at a scan rate of 10 °C/min; Col$_h$: hexagonal columnar. Value in parentheses represents the change in enthalpy (ΔH). The Col$_h^{I}$→Col$_h^{II}$ phase-transition temperature was determined from the PXRD data in **d** and **e**. **b**,**c**, POM micrographs of ^{Im}TP[Dy]$_{2.0}$ at 25 °C upon cooling from 190 °C (**b**), and at 25 °C after ageing for 8 h (**c**); scale bars, 100 μm. **d**,**e**, Variable-temperature PXRD patterns for the q = 0.6–6 nm^{-1} (**d**) and q = 6–20 nm^{-1} (**e**) regions of a bulk sample of ^{Im}TP[Dy]$_{2.0}$, measured upon heating and subsequent cooling (heating/cooling rate: 10 °C/min); a.u., arbitrary unit. Indices of reflections are shown in the PXRD patterns.

Supplementary Table 3 | XRD data for a bulk sample of $^{Im}TP[Dy]_{2.0}$ upon heating

T (°C)	*q* (nm^{-1})	$d_{obs.}$ (nm)	$d_{calc.}$ (nm)	*hkl*
57	1.89	3.33	3.31	100
(Col_h^{I})*	3.28	1.92	1.91	110
	3.80	1.66	1.66	200
147	2.15	2.92	2.90	100
(Col_h^{II})†	3.75	1.68	1.67	110
	4.33	1.45	1.45	200
	6.51	0.97	0.97	300
	7.84	0.80	0.80	310
	17.57	0.36	0.36	001

**P6mm* hexagonal cell parameter at 57 °C: a = 3.83 nm; †*P6mm* hexagonal cell parameters at 147 °C: a = 3.35 nm and c = 0.36 nm.

$^{Im}TP[Dy]_{0.6}$

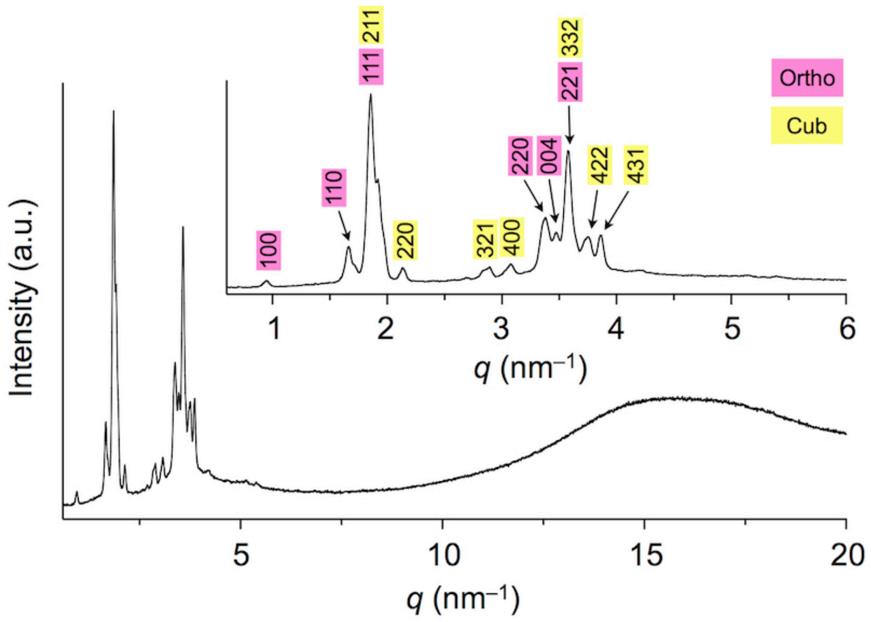

Supplementary Figure 7 | PXRD pattern and magnification of the small-angle region (*q* = 0.6–6 nm^{-1}) of a bulk sample of $^{Im}TP[Dy]_{0.6}$ at 27 °C. The peaks could not be fully assigned, as $^{Im}TP[Dy]_{0.6}$ consists of a mixture of domains of the Cub and Ortho phases; a.u., arbitrary unit. Indices of the reflections for the Cub (yellow) and Ortho (magenta) phases are shown in the magnification of the PXRD pattern.

Thermally processed ^{Im}TP[Dy]$_{0.75}$ in the presence of a 10-T magnetic field

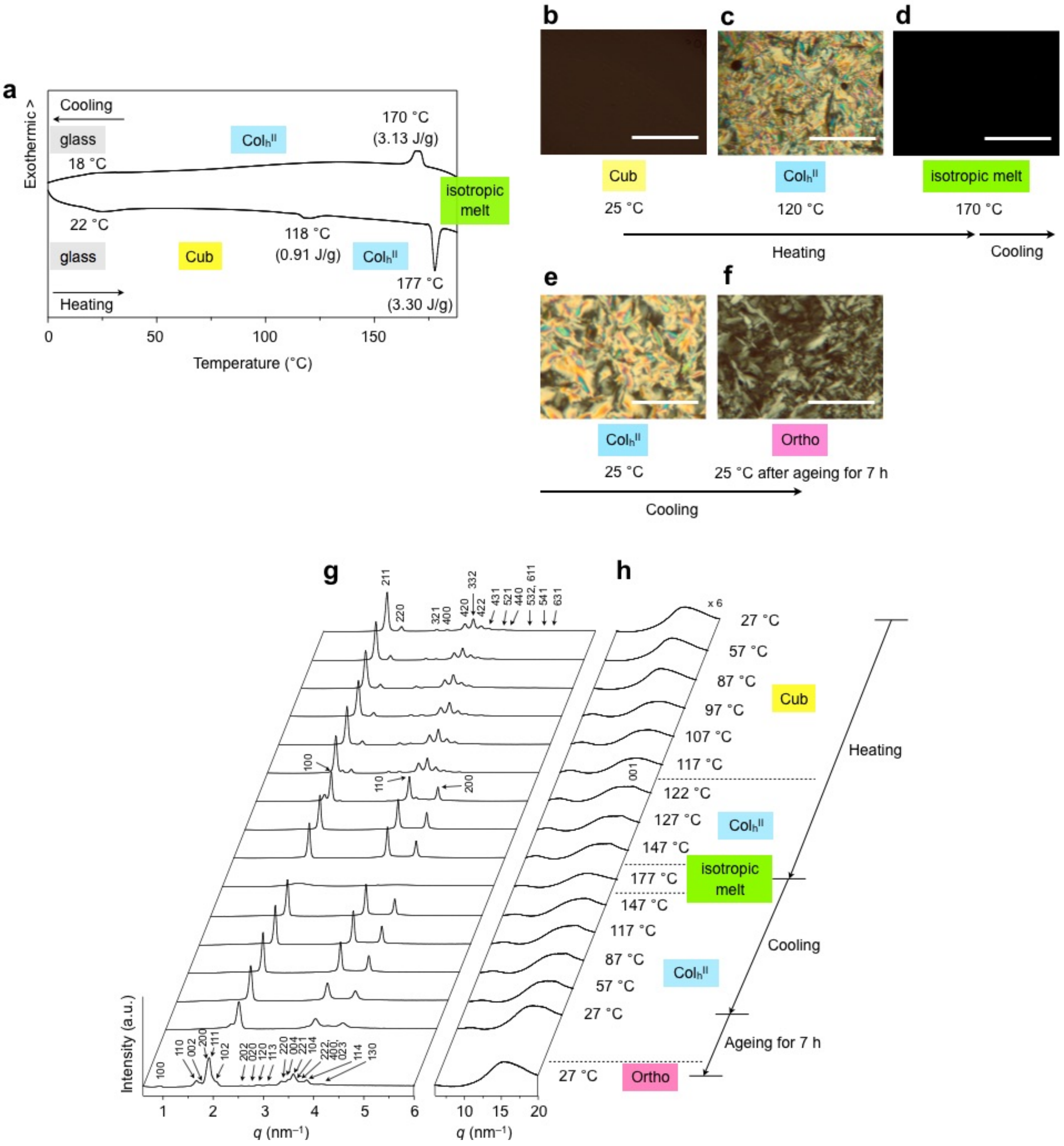

Supplementary Figure 8 | Structural characterization and phase behaviour of thermally processed ^{Im}TP[Dy]$_{0.75}$ in the presence of a 10-T magnetic field. **a**, DSC profile (first heating/cooling cycle) of thermally processed ^{Im}TP[Dy]$_{0.75}$ in the presence of a 10-T magnetic field, measured at a scan rate of 10 °C/min; Cub: cubic, Col$_h$: hexagonal columnar. Values in parentheses represent the changes in enthalpy (ΔH). **b**–**f**, POM micrographs of thermally processed ^{Im}TP[Dy]$_{0.75}$ in the presence of a 10-T magnetic field at 25 °C (**b**), 120 °C (**c**) and 170 °C (**d**) upon heating, and at 25 °C upon cooling from 180 °C (**e**), and at 25 °C after ageing for 7 h (**f**); Ortho: orthorhombic; scale bars, 100 µm. **g**,**h**, Variable-temperature PXRD patterns for the q = 0.6–6 nm^{-1} (**g**) and q = 6–20 nm^{-1} (**h**) regions of a bulk sample of thermally processed ^{Im}TP[Dy]$_{0.75}$ in the presence of a 10-T magnetic field, measured upon heating and subsequent cooling (heating/cooling rate: 10 °C/min); a.u., arbitrary unit. Indices of reflections are shown in the PXRD patterns.

Supplementary Table 4 | XRD data for a bulk sample of thermally processed ^{Im}TP[Dy]$_{0.75}$ in the presence of a 10-T magnetic field upon heating

T (°C)	q (nm^{-1})	$d_{obs.}$ (nm)	$d_{calc.}$ (nm)	*hkl*
27	1.85	3.39	3.37	211
(Cub)*	2.14	2.94	2.92	220
	2.85	2.21	2.21	321
	3.05	2.06	2.07	400
	3.41	1.85	1.85	420
	3.57	1.76	1.76	332
	3.73	1.69	1.69	422
	3.87	1.62	1.62	431
	4.18	1.51	1.51	521
	4.30	1.46	1.46	440
	4.67	1.35	1.34	532
	4.67	1.35	1.34	611
	4.94	1.27	1.28	541
	5.18	1.21	1.22	631
147	2.11	2.98	2.96	100
(Col$_h^{II}$)†	3.67	1.71	1.71	110
	4.25	1.48	1.48	200
	17.55	0.36	0.36	001

*$Ia\bar{3}d$ cubic cell parameter at 27 °C: a = 8.26 nm; †$P6mm$ hexagonal cell parameters at 147 °C: a = 3.42 nm and c = 0.36 nm.

Supplementary Table 5 | XRD data for a bulk sample of thermally processed $^{Im}TP[Dy]_{0.75}$ in the presence of a 10-T magnetic field upon cooling from the isotropic melt

T (°C)	q (nm^{-1})	$d_{obs.}$ (nm)	$d_{calc.}$ (nm)	*hkl*
27*	0.95	6.60	6.60	100
(Ortho)†	1.67	3.76	3.78	110
	1.71	3.67	3.60	002
	1.90	3.32	3.30	200
	1.92	3.28	3.35	111
	2.02	3.12	3.16	102
	2.55	2.46	2.43	202
	2.71	2.32	2.31	020
	2.89	2.18	2.18	120
	3.10	2.03	2.03	113
	3.36	1.87	1.89	220
	3.50	1.80	1.80	004
	3.59	1.75	1.83	221
	3.63	1.73	1.74	104
	3.77	1.67	1.67	222
	3.77	1.67	1.66	023
	3.77	1.67	1.65	400
	3.85	1.63	1.63	114
	4.14	1.52	1.50	130

*The sample was aged for 7 h at 27 °C; †*Pbcm* orthorhombic cell parameters at 27 °C: a = 6.60 nm, b = 4.61 nm and c = 7.20 nm.

^{Im}TP[La]$_{2.0}$

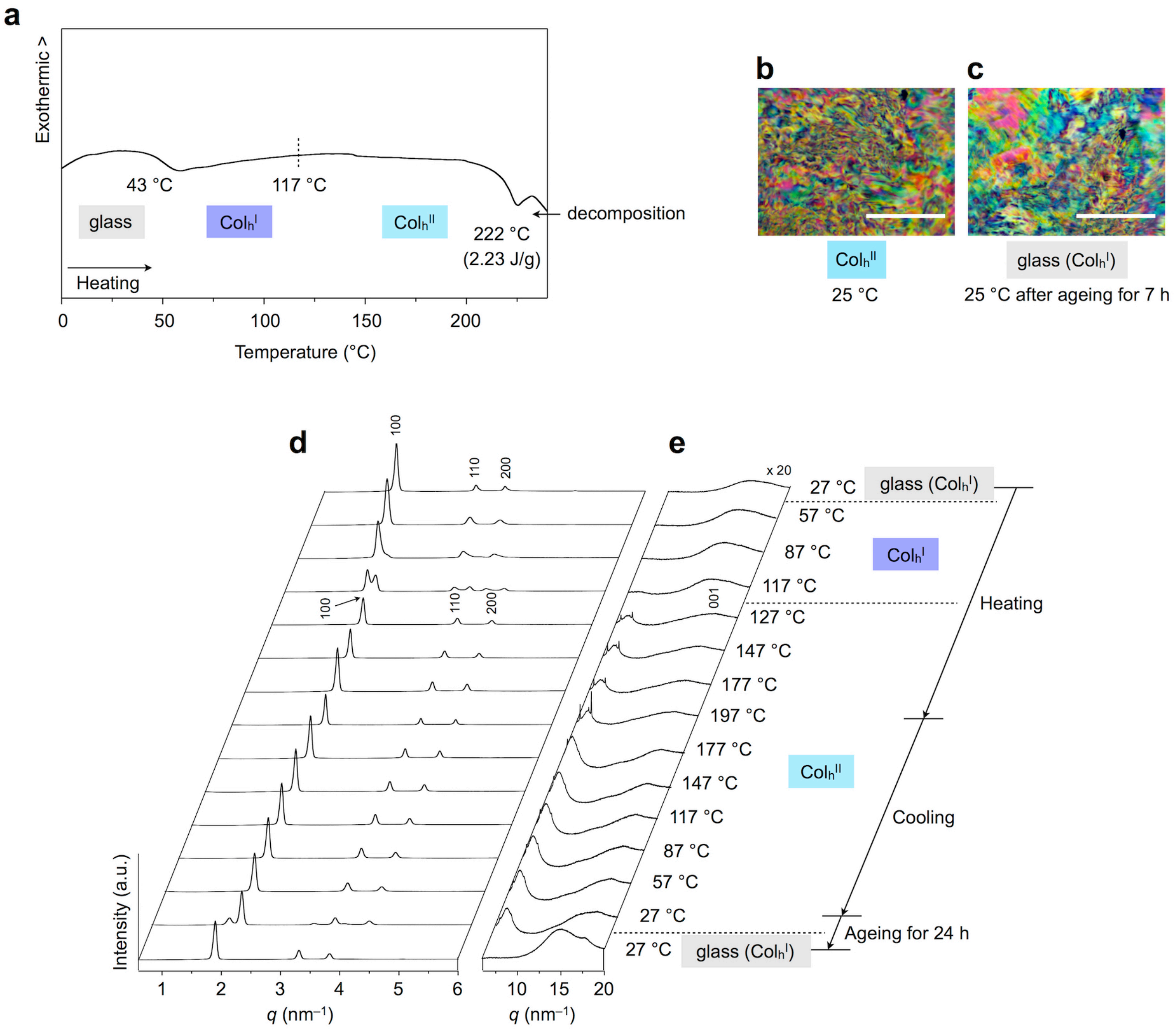

Supplementary Figure 9 | Structural characterization and phase behaviour of thermally processed ^{Im}TP[La]$_{2.0}$ in the absence of a magnetic field. **a**, DSC profile (first heating) of ^{Im}TP[La]$_{2.0}$, measured at a scan rate of 10 °C/min; Col$_h$: hexagonal columnar. Value in parentheses represents the change in enthalpy (ΔH). The Col$_h^I$→Col$_h^{II}$ phase-transition temperature was determined from the PXRD data in **d** and **e**. **b**,**c**, POM micrographs of ^{Im}TP[La]$_{2.0}$ at 25 °C upon cooling from 190 °C (**b**), and at 25 °C after ageing for 7 h (**c**); scale bars, 100 µm. **d**,**e**, Variable-temperature PXRD patterns for the q = 0.6–6 nm^{-1} (**d**) and q = 6–20 nm^{-1} (**e**) regions of a bulk sample of ^{Im}TP[La]$_{2.0}$, measured upon heating and subsequent cooling (heating/cooling rate: 10 °C/min); a.u., arbitrary unit. Indices of the reflections are shown in the PXRD patterns.

Supplementary Table 6 | XRD data for a bulk sample of $^{Im}TP[La]_{2.0}$ upon heating

T (°C)	q (nm^{-1})	$d_{obs.}$ (nm)	$d_{calc.}$ (nm)	*hkl*
57	1.87	3.36	3.37	100
(Col_h^{I})*	3.27	1.92	1.93	110
	3.79	1.66	1.67	200
	4.97	1.26	1.26	210
147	2.15	2.92	2.90	100
(Col_h^{II})†	3.74	1.68	1.67	110
	4.33	1.46	1.45	200
	6.52	0.97	0.97	300
	17.61	0.36	0.36	001

**P6mm* hexagonal cell parameter at 57 °C: a = 3.85 nm; †*P6mm* hexagonal cell parameters at 147 °C: a = 3.35 nm and c = 0.36 nm.

^{Im}TP[La]$_{0.75}$ and ^{Im}TP[La]$_{1.0}$

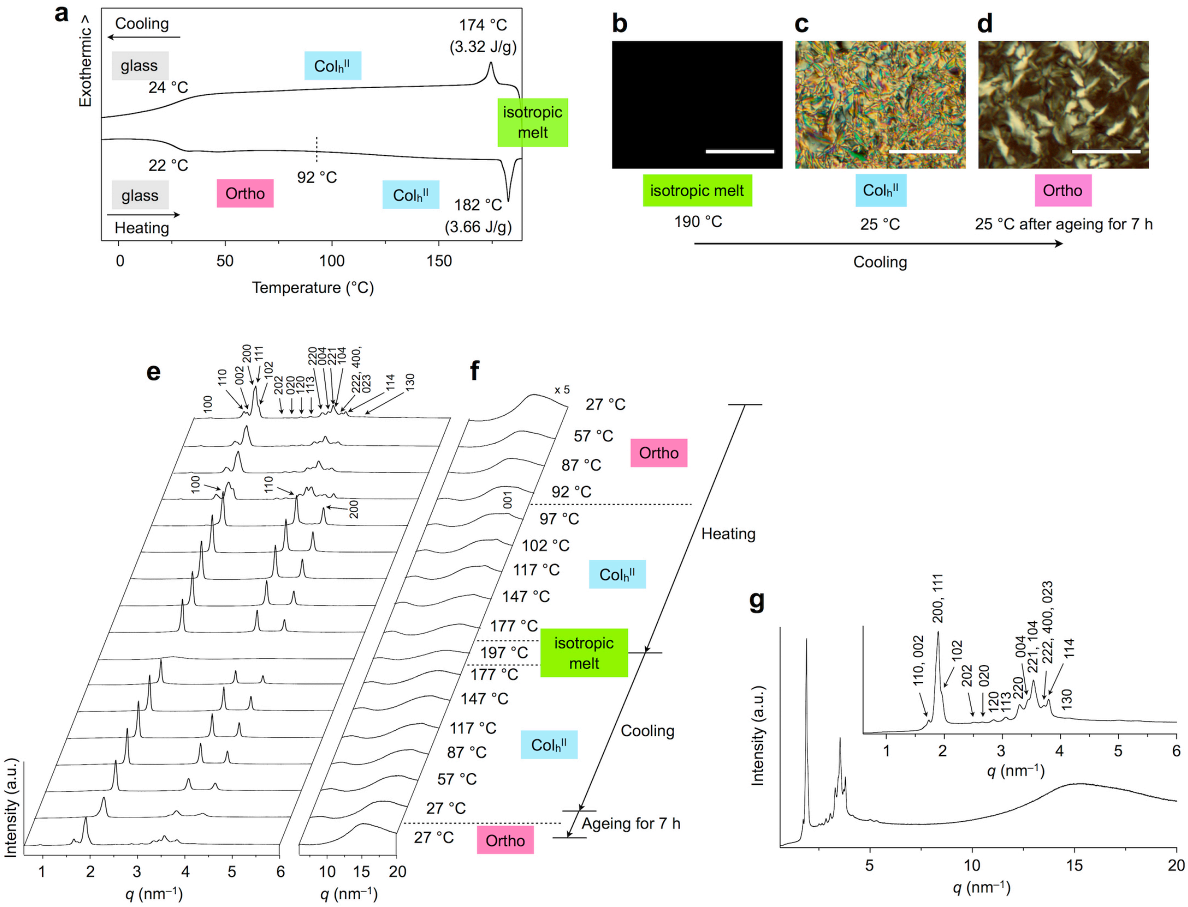

Supplementary Figure 10 | Structural characterization and phase behaviour of thermally processed ^{Im}TP[La]$_{0.75}$ and ^{Im}TP[La]$_{1.0}$ in the absence of a magnetic field. a, DSC profile (second heating/cooling cycle) of ^{Im}TP[La]$_{0.75}$, measured at a scan rate of 10 °C/min; Ortho: orthorhombic, Col$_h$: hexagonal columnar. Values in parentheses represent the changes in enthalpy (ΔH). The Ortho→Col$_h$^{II} phase-transition temperature was determined from the PXRD data in **e** and **f**. **b–d,** POM micrographs of ^{Im}TP[La]$_{0.75}$ at 190 °C (**b**) and 25 °C (**c**) upon cooling from 195 °C, and at 25 °C after ageing for 7 h (**d**); scale bars, 100 µm. **e**,**f**, Variable-temperature PXRD patterns for the q = 0.6–6 nm^{-1} (**e**) and q = 6–20 nm^{-1} (**f**) regions of a bulk sample of ^{Im}TP[La]$_{0.75}$, measured upon sequential heating and cooling (heating/cooling rate: 10 °C/min). **g**, PXRD pattern and magnification of the small-angle region (scattering vector q = 0.6–6 nm^{-1}) of a bulk sample of ^{Im}TP[La]$_{1.0}$ at 27 °C; a.u., arbitrary unit. Indices of the reflections are shown in the PXRD patterns.

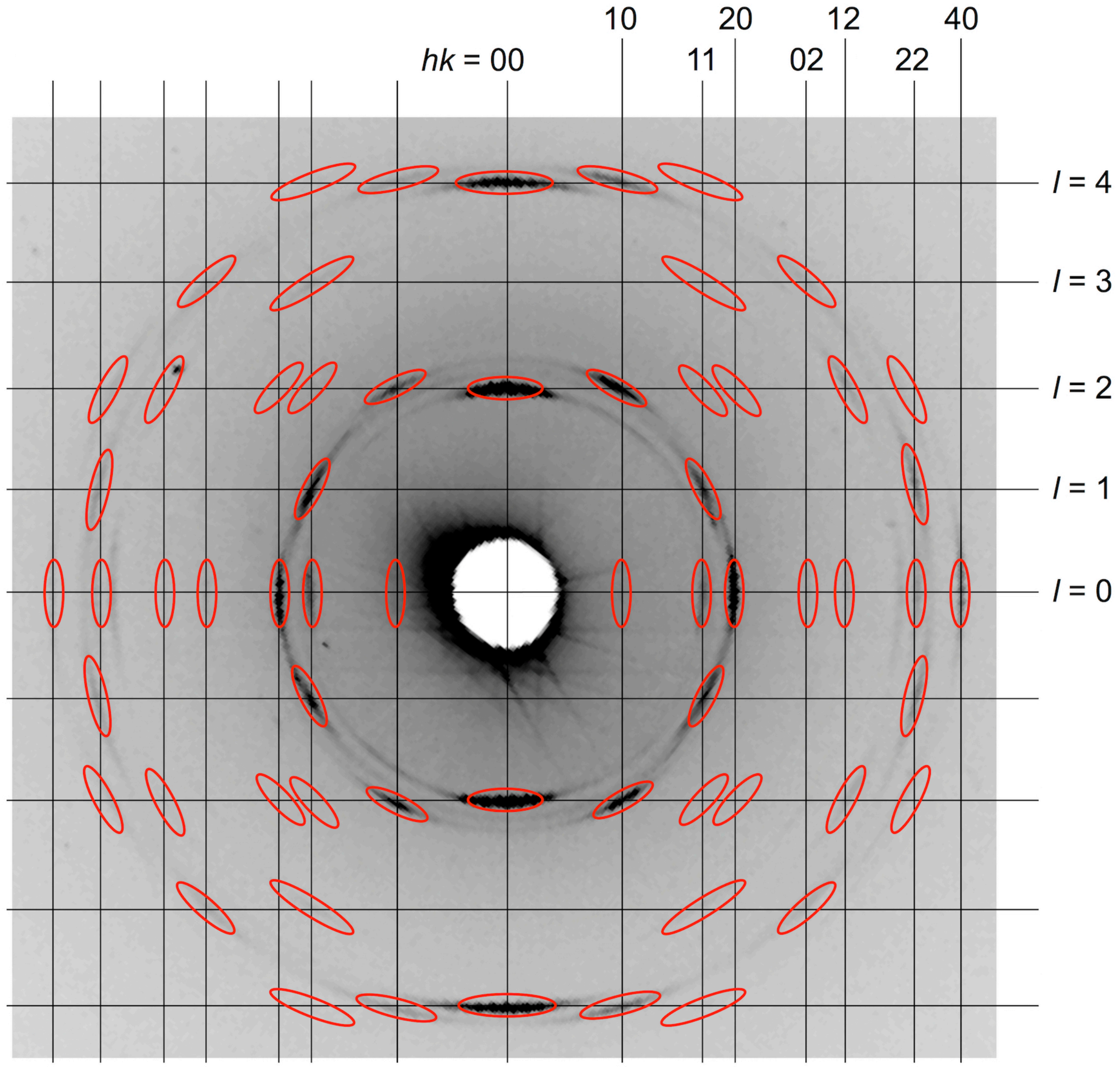

Supplementary Figure 11 | Through-view XRD image observed at 25 °C for a shear-oriented film of the Ortho phase of ^{Im}TP[La]$_{0.75}$ on a sapphire substrate. *h*, *k* and *l* represent the indices of reflections. The diffraction arcs in an equatorial direction with *d*-spacings of 3.8, 3.4, 1.9 and 1.7 nm were assigned to diffractions from the (110), (200), (220) and (400) planes of a 2D rectangular structure, respectively. The two intense arcs in the meridional direction with *d*-spacings of 3.7 and 1.8 nm were assigned to the diffractions from the (002) and (004) planes of a 1D periodic (layer) structure, respectively, which develops perpendicular to the 2D rectangular lattice. All diffractions observed in the XRD image could be fully indexed by considering a *Pbcm* lattice with parameters *a* = 6.8 nm, *b* = 4.7 nm and *c* = 7.4 nm.

Supplementary Table 7 | XRD data for a bulk sample of $^{Im}TP[La]_{0.75}$ upon heating

T (°C)	q (nm^{-1})	$d_{obs.}$ (nm)	$d_{calc.}$ (nm)	*hkl*
27	0.94	6.67	6.75	100
(Ortho)*	1.64	3.83	3.83	110
	1.70	3.69	3.68	002
	1.87	3.37	3.37	200
	1.88	3.33	3.40	111
	1.95	3.23	3.23	102
	2.50	2.51	2.49	202
	2.65	2.38	2.33	020
	2.84	2.21	2.20	120
	3.04	2.07	2.07	113
	3.28	1.91	1.92	220
	3.42	1.84	1.84	004
	3.52	1.79	1.85	221
	3.55	1.77	1.78	104
	3.70	1.70	1.70	222
	3.70	1.70	1.69	400
	3.70	1.70	1.69	023
	3.78	1.66	1.66	114
	4.14	1.52	1.51	130
147	2.13	2.95	2.95	100
(Col_h^{II})†	3.69	1.70	1.70	110
	4.27	1.47	1.47	200
	17.64	0.36	0.36	001

**Pbcm* orthorhombic cell parameters at 27 °C: a = 6.75 nm, b = 4.66 nm and c = 7.37 nm; †*P6mm* hexagonal cell parameters at 147 °C: a = 3.40 nm and c = 0.36 nm.

^{Im}TP[La]$_{0.5}$

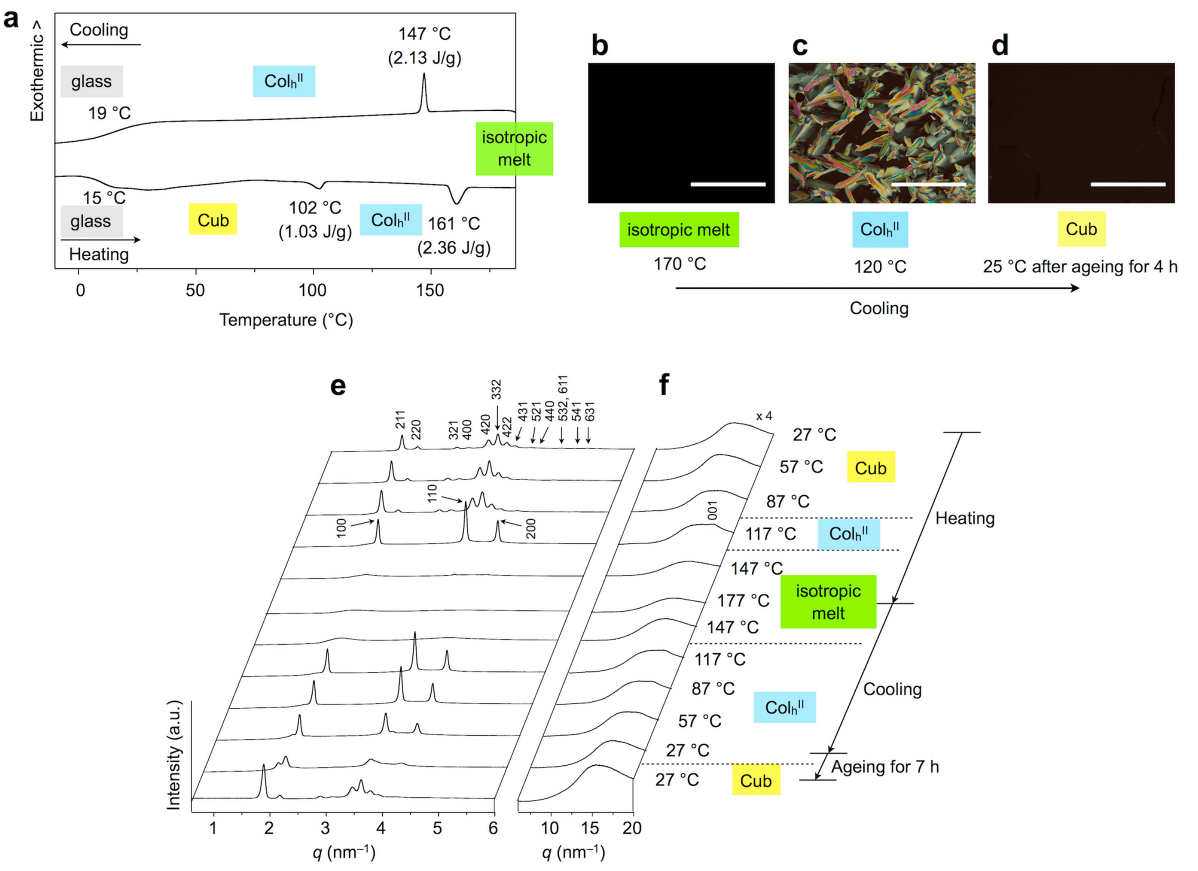

Supplementary Figure 12 | Structural characterization and phase behaviour of thermally processed ^{Im}TP[La]$_{0.5}$ in the absence of a magnetic field. **a**, DSC profile (second heating/cooling cycle) of ^{Im}TP[La]$_{0.5}$, measured at a scan rate of 10 °C/min; Cub: cubic, Col_h: hexagonal columnar. Values in parentheses represent the changes in enthalpy (ΔH). **b**–**d**, POM micrographs of ^{Im}TP[La]$_{0.5}$ at 170 °C (**b**) and 120 °C (**c**) upon cooling from 180 °C, and at 25 °C after ageing for 4 h (**d**); scale bars, 100 μm. **e**,**f**, Variable-temperature PXRD patterns for the q = 0.6–6 nm^{-1} (**e**) and q = 6–20 nm^{-1} (**f**) regions of a bulk sample of ^{Im}TP[La]$_{0.5}$, measured upon heating and subsequent cooling (heating/cooling rate: 10 °C/min); a.u., arbitrary unit. Indices of the reflections are shown in the PXRD patterns.

Supplementary Table 8 | XRD data for a bulk sample of $^{Im}TP[La]_{0.5}$ upon heating

T (°C)	q (nm^{-1})	$d_{obs.}$ (nm)	$d_{calc.}$ (nm)	*hkl*
27	1.87	3.37	3.36	211
(Cub)*	2.15	2.92	2.91	220
	2.85	2.21	2.20	321
	3.04	2.07	2.06	400
	3.41	1.85	1.84	420
	3.57	1.76	1.76	332
	3.73	1.69	1.68	422
	3.88	1.62	1.62	431
	4.16	1.51	1.50	521
	4.31	1.46	1.46	440
	4.74	1.33	1.34	532
	4.74	1.33	1.34	611
	4.95	1.27	1.27	541
	5.17	1.22	1.21	631
117	2.12	3.01	3.00	100
(Col_h^{II})†	3.67	1.74	1.73	110
	4.25	1.50	1.50	200
	17.57	0.36	0.36	001

*$Ia\bar{3}d$ cubic cell parameter at 27 °C: a = 8.24 nm; †$P6mm$ hexagonal cell parameters at 117 °C: a = 3.47 nm and c = 0.36 nm.

Thermally processed ^{Im}TP[La]$_{0.75}$ in the presence of a magnetic field

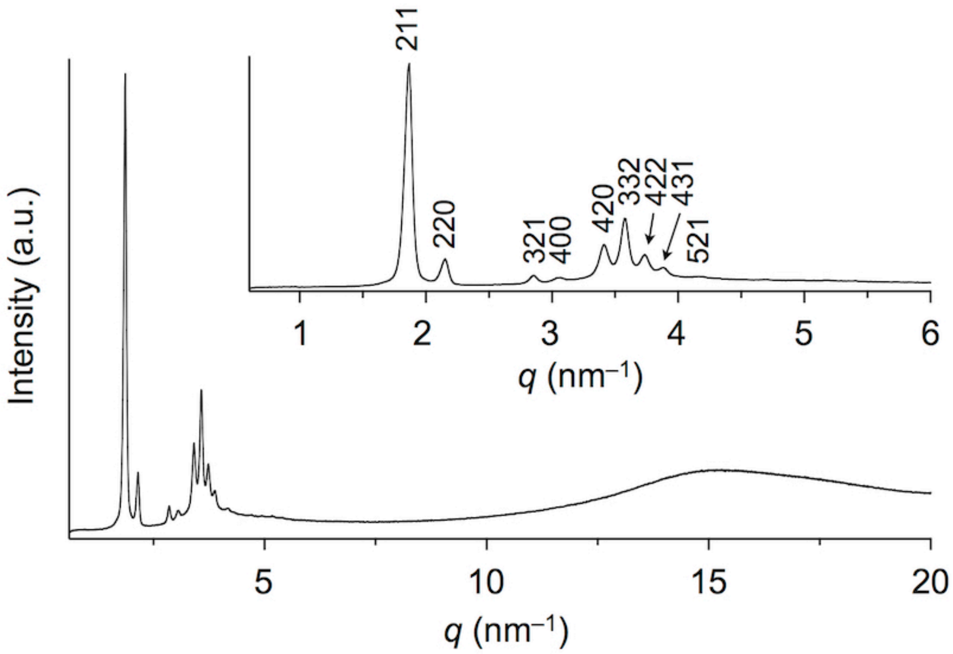

Supplementary Figure 13 | PXRD pattern of ^{Im}TP[La]$_{0.75}$. PXRD pattern and magnification of the small-angle region (scattering vector q = 0.6–6 nm^{-1}) of a bulk sample of ^{Im}TP[La]$_{0.75}$ at 27 °C, after heating to 180 °C and subsequent cooling to 25 °C in the presence of a 10-T magnetic field; a.u., arbitrary unit. Indices of the reflections are shown in the magnification of the PXRD pattern.

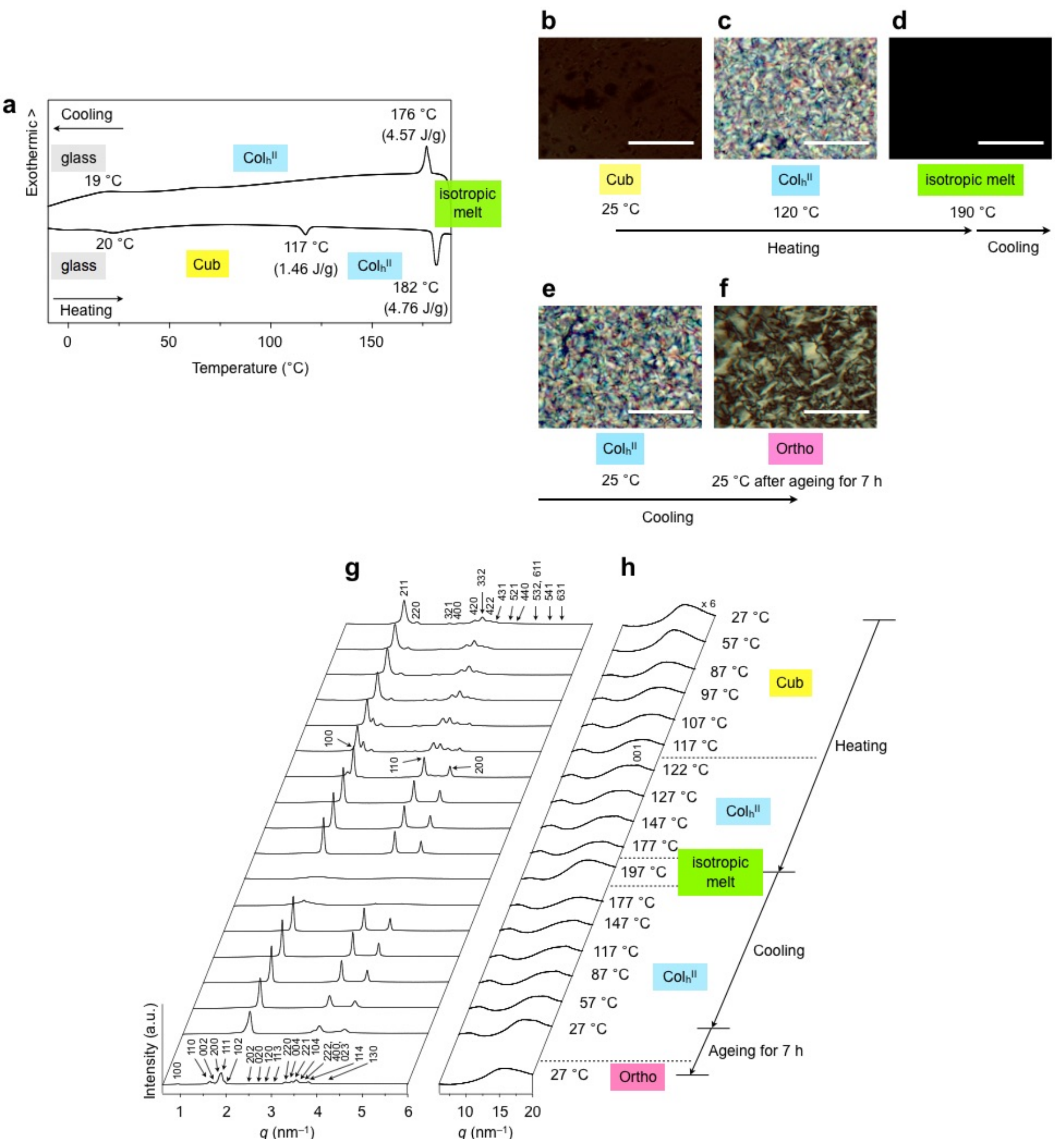

Supplementary Figure 14 | Structural characterization and phase behaviour of thermally processed ^{Im}TP[La]$_{0.75}$ in the presence of a 10-T magnetic field. **a**, DSC profile (first heating/cooling cycle) of thermally processed ^{Im}TP[La]$_{0.75}$ in the presence of a 10-T magnetic field, measured at a scan rate of 10 °C/min; Cub: cubic, Col$_h$: hexagonal columnar. Values in parentheses represent the changes in enthalpy (ΔH). **b**–**f**, POM micrographs of thermally processed ^{Im}TP[La]$_{0.75}$ in the presence of a 10-T magnetic field at 25 °C (**b**), 120 °C (**c**) and 190 °C (**d**) upon heating, and at 25 °C upon cooling from 195 °C (**e**), and at 25 °C after ageing for 7 h (**f**); Ortho: orthorhombic; scale bars, 100 µm. **g**,**h**, Variable-temperature PXRD patterns for the q = 0.6–6 nm^{-1} (**g**) and q = 6–20 nm^{-1} (**h**) regions of a bulk sample of thermally processed ^{Im}TP[La]$_{0.75}$ in the presence of a 10-T magnetic field, measured upon heating and subsequent cooling (heating/cooling rate: 10 °C/min); a.u., arbitrary unit. Indices of the reflections are shown in the PXRD patterns.

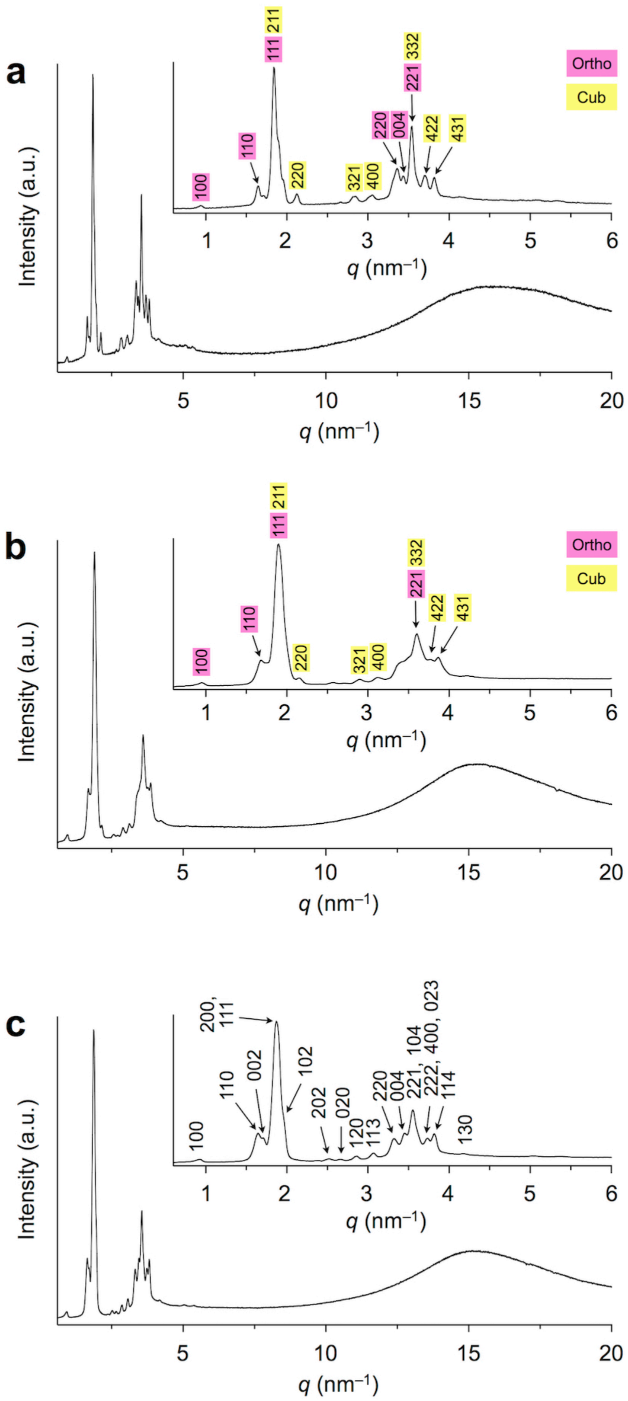

Supplementary Figure 15 | PXRD patterns of ^{Im}TP[La]$_{0.75}$. **a–c**, PXRD patterns and magnifications of the small-angle region (scattering vector q = 0.6–6 nm^{-1}) of bulk samples of thermally processed ^{Im}TP[La]$_{0.75}$ in the presence of a 5-T magnetic field, measured at 27 °C (**a**) and ^{Im}TP[La]$_{0.75}$ at 27 °C, after being thermally processed at 150 °C (**b**) and at 90 °C (**c**) in the presence of a 10-T magnetic field; a.u., arbitrary unit. The peaks in **a** and **b** could not be fully assigned, since the bulk sample of ^{Im}TP[La]$_{0.75}$ consists of a mixture of domains of the Cub (yellow) and Ortho (magenta) structures. Indices of the reflections are shown in the magnifications of the PXRD patterns.

Supplementary Table 9 | XRD data for a bulk sample of thermally processed $^{Im}TP[La]_{0.75}$ in the presence of a 10-T magnetic field upon heating

T (°C)	*q* (nm^{-1})	$d_{obs.}$ (nm)	$d_{calc.}$ (nm)	*hkl*
27	1.85	3.39	3.38	211
(Cub)*	2.14	2.94	2.93	220
	2.84	2.21	2.21	321
	3.03	2.07	2.07	400
	3.40	1.85	1.85	420
	3.56	1.77	1.77	332
	3.71	1.70	1.69	422
	3.86	1.63	1.63	431
	4.14	1.52	1.51	521
	4.29	1.47	1.46	440
	4.68	1.34	1.34	532
	4.68	1.34	1.34	611
	4.93	1.28	1.28	541
	5.15	1.22	1.22	631
147	2.13	2.95	2.95	100
(Col_h^{II})†	3.69	1.70	1.70	110
	4.27	1.47	1.47	200
	17.53	0.36	0.36	001

*$Ia\bar{3}d$ cubic cell parameter at 27 °C: *a* = 8.28 nm; †*P*6*mm* hexagonal cell parameters at 147 °C: *a* = 3.40 nm and *c* = 0.36 nm.

Supplementary Table 10 | XRD data for a bulk sample of thermally processed $^{Im}TP[La]_{0.75}$ in the presence of a 10-T magnetic field upon cooling from the isotropic melt

T (°C)	*q* (nm^{-1})	$d_{obs.}$ (nm)	$d_{calc.}$ (nm)	*hkl*
27*	0.94	6.67	6.69	100
(Ortho)†	1.64	3.83	3.81	110
	1.70	3.69	3.66	002
	1.87	3.35	3.34	200
	1.90	3.32	3.38	111
	1.95	3.23	3.21	102
	2.52	2.49	2.47	202
	2.66	2.37	2.32	020
	2.86	2.20	2.19	120
	3.05	2.06	2.05	113
	3.30	1.90	1.91	220
	3.44	1.83	1.83	004
	3.54	1.78	1.85	221
	3.58	1.76	1.76	104
	3.73	1.69	1.69	222
	3.73	1.69	1.68	023
	3.73	1.69	1.67	400
	3.80	1.65	1.65	114
	4.14	1.52	1.51	130

*The sample was aged for 7 h at 27 °C; †*Pbcm* orthorhombic cell parameters at 27 °C: a = 6.69 nm, b = 4.64 nm and c = 7.32 nm.

^{Im}TPBr$_6$

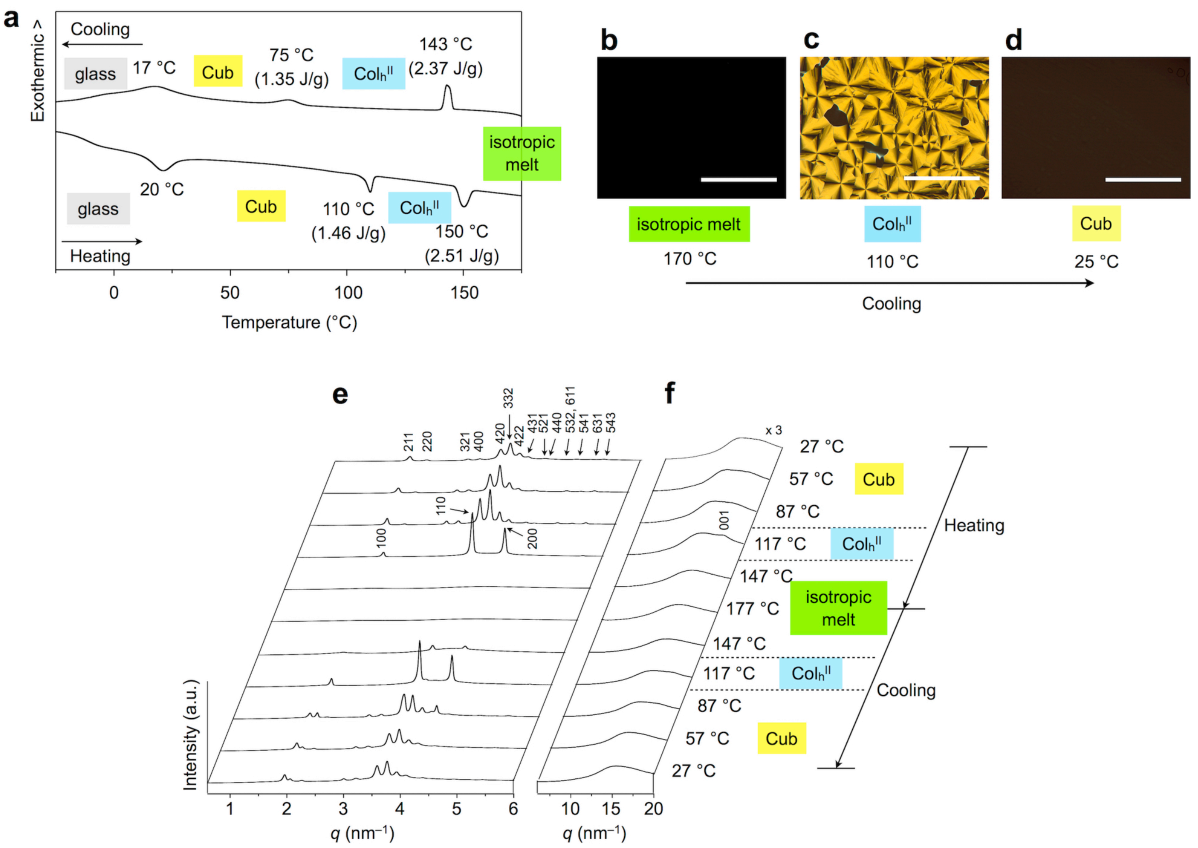

Supplementary Figure 16 | Structural characterization and phase behaviour of thermally processed ^{Im}TPBr$_6$ in the absence of a magnetic field. **a**, DSC profile (second heating/cooling cycle) of ^{Im}TPBr$_6$, measured at a scan rate of 10 °C/min; Cub: cubic, Col$_h$: hexagonal columnar. Values in parentheses represent the changes in enthalpy (ΔH). **b–d**, POM micrographs of ^{Im}TPBr$_6$ at 170 °C (**b**), 110 °C (**c**) and 25 °C (**d**) upon cooling from 180 °C; scale bars, 100 µm. **e**,**f**, Variable-temperature PXRD patterns for the q = 0.6–6 nm^{-1} (**e**) and q = 6–20 nm^{-1} (**f**) regions of a bulk sample of ^{Im}TPBr$_6$, measured upon heating and subsequent cooling (heating/cooling rate: 10 °C/min); a.u., arbitrary unit. Indices of the reflections are shown in the PXRD patterns.

Supplementary Table 11 | XRD data for a bulk sample of $^{Im}TPBr_6$ upon heating

T (°C)	q (nm^{-1})	$d_{obs.}$ (nm)	$d_{calc.}$ (nm)	hkl
27	1.91	3.28	3.27	211
(Cub)*	2.21	2.84	2.83	220
	2.94	2.14	2.14	321
	3.14	2.00	2.00	400
	3.52	1.79	1.79	420
	3.68	1.71	1.71	332
	3.84	1.64	1.63	422
	3.99	1.57	1.57	431
	4.30	1.46	1.46	521
	4.42	1.42	1.41	440
	4.86	1.30	1.30	532
	4.86	1.30	1.30	611
	5.09	1.24	1.24	541
	5.33	1.18	1.18	631
	5.56	1.13	1.13	543
117	2.12	2.97	2.98	100
(Col_h^{II})†	3.69	1.70	1.72	110
	4.26	1.48	1.49	200
	5.55	1.13	1.13	210
	17.64	0.36	0.36	001

*$Ia\bar{3}d$ cubic cell parameter at 27 °C: a = 8.00 nm; †$P6mm$ hexagonal cell parameters at 117 °C: a = 3.45 nm and c = 0.36 nm.

Experimental Setups for Thermal Processing, *in situ* POM and *in situ* X-ray Diffraction

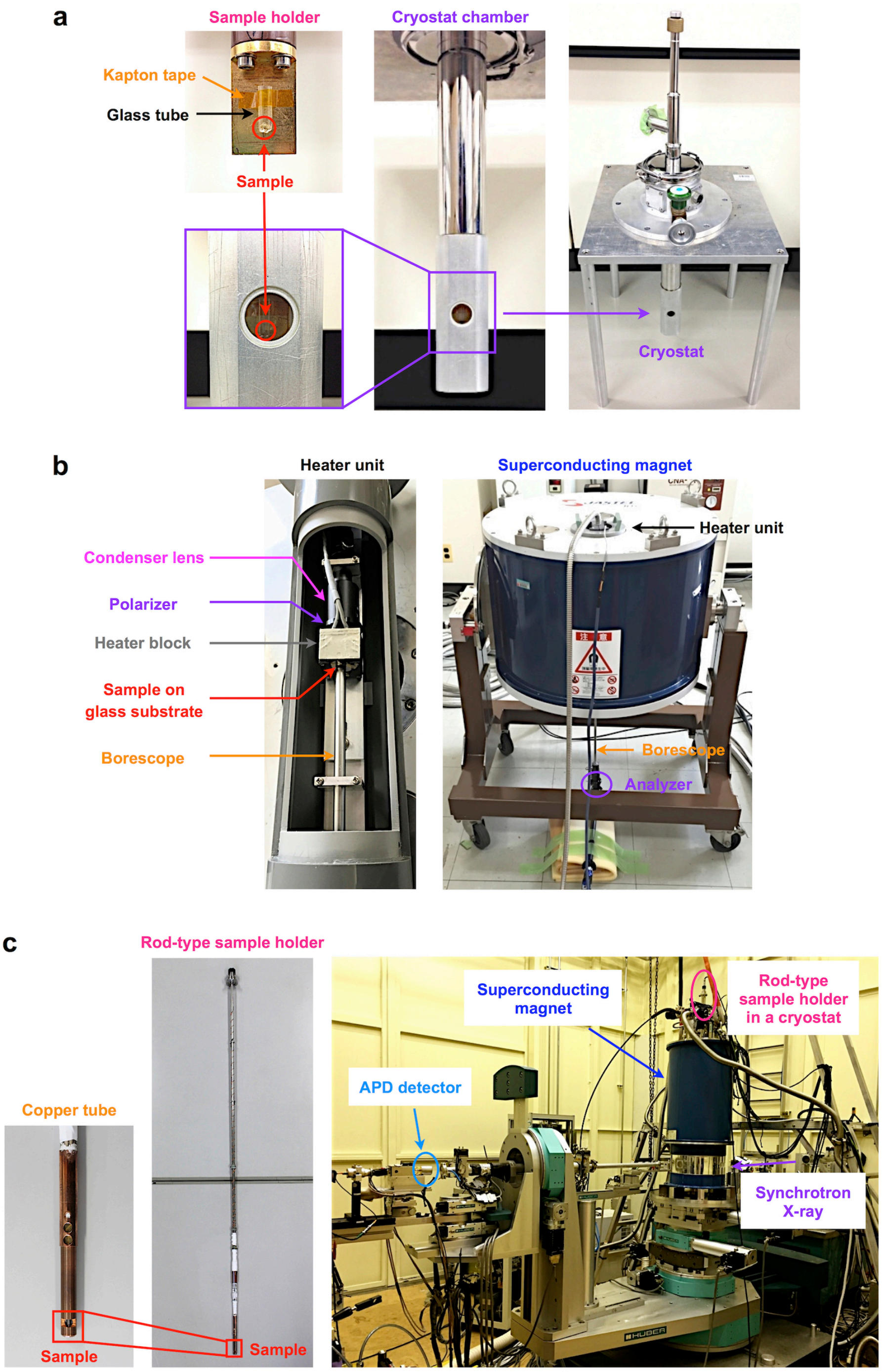

Supplementary Figure 17 | Experimental setups for thermal processing, *in situ* POM and *in situ* X-ray diffraction. a, Experimental setup for thermal processing of $^{Im}TP[Ln]_x$ in a 10-T magnet. **b**, Experimental setup for *in situ* POM of a film sample of $^{Im}TP[La]_{0.75}$ in a 10-T magnet. **c**, Experimental setup for *in situ* X-ray diffraction experiments on a bulk sample of $^{Im}TP[La]_{0.75}$ in the presence of a magnetic field (7 T) in an 8-T superconducting magnet.

In situ X-ray Diffraction Data in a 7-T Magnetic Field

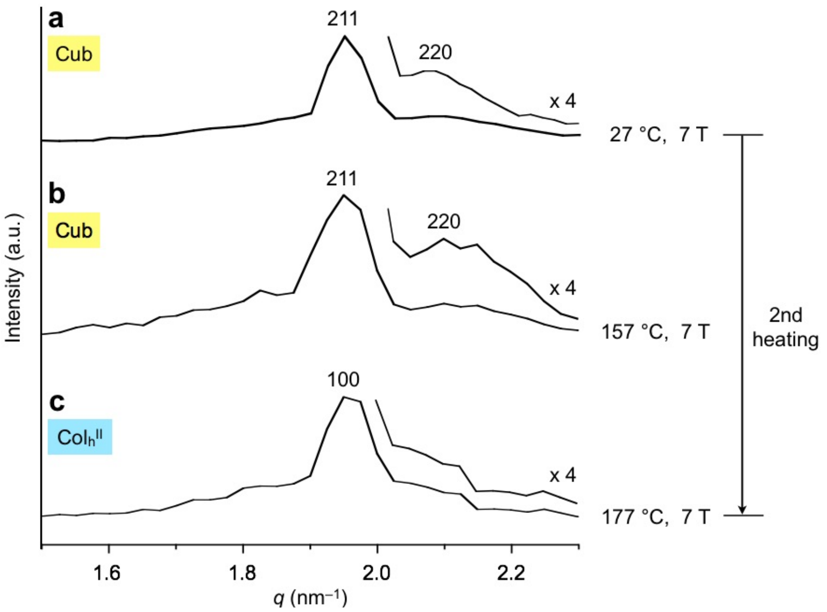

Supplementary Figure 18 | *In situ* X-ray diffraction observation in a 7-T magnetic field. a–c, X-ray diffraction patterns of the small-angle region (scattering vector q = 1.5–2.3 nm^{-1}) of a bulk sample of ^{Im}TP[La]$_{0.75}$ in a glass capillary (diameter: 2.5 mm) after thermal processing in a 7-T magnetic field. The diffraction patterns were measured at 27 (**a**), 157 (**b**) and 177 °C (**c**) upon second heating (heating rate: 20 °C/min) in a 7-T magnetic field. Indices of the reflections are shown at the top of the X-ray diffraction peaks.

FIR Spectra

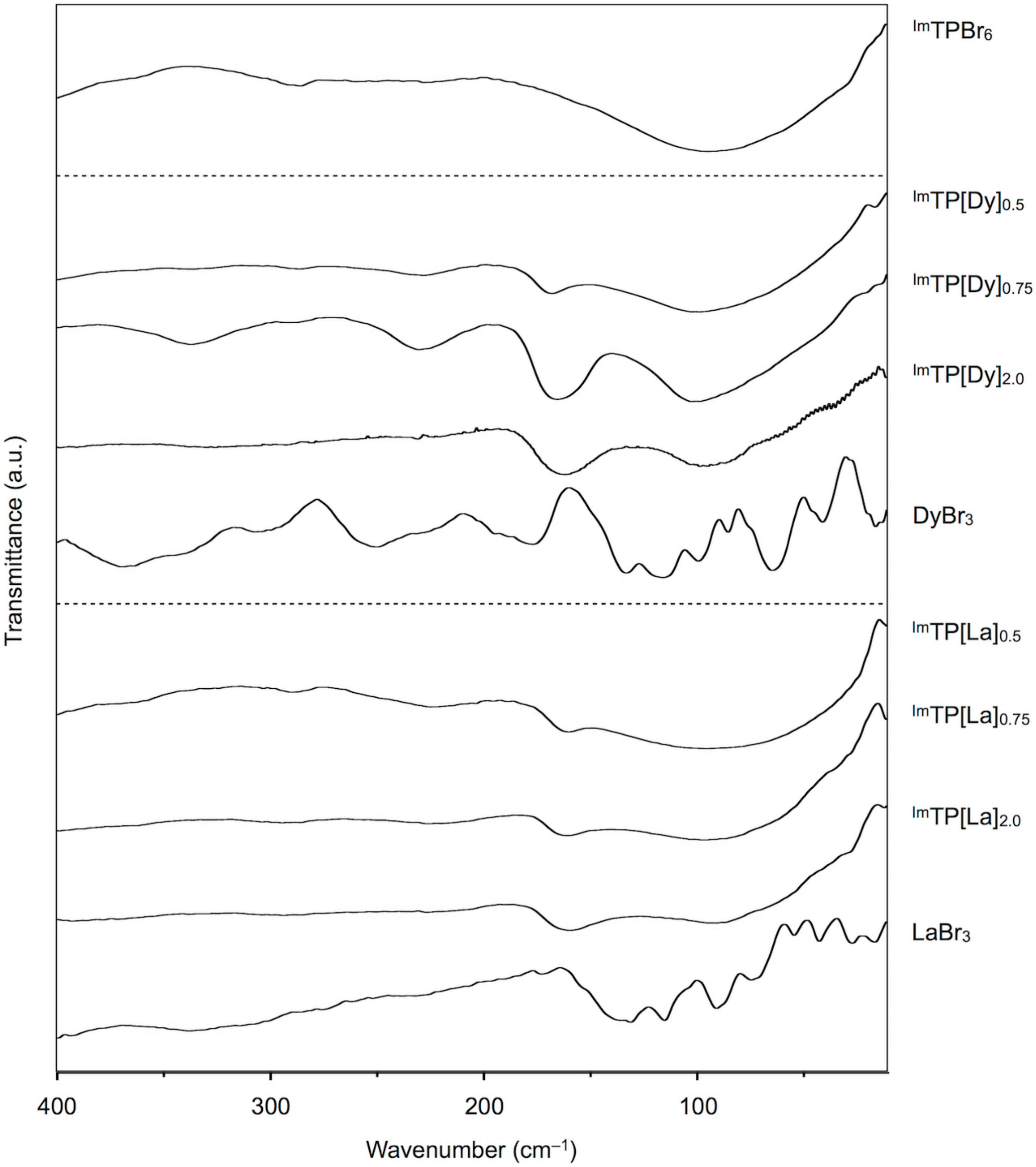

Supplementary Figure 19 | FIR spectra at 25 °C of $^{Im}TPBr_6$, $^{Im}TP[Dy]_x$ (x = 0.5, 0.75 and 2.0), $DyBr_3$, $^{Im}TP[La]_x$ (x = 0.5, 0.75 and 2.0) and $LaBr_3$ (a.u., arbitrary unit).

Supplementary References

1. Motoyanagi, J., Fukushima, T. & Aida, T. Discotic liquid crystals stabilized by interionic interactions: imidazolium ion-anchored paraffinic triphenylene. *Chem. Commun.* 101–103 (2005).
2. Alam, M. A. *et al.* "Bicontinuous cubic" liquid crystalline materials from discotic molecules: a special effect of paraffinic side chains with ionic liquid pendants. *J. Am. Chem. Soc.* **131,** 17722–17723 (2009).
3. Choca, M., Ferraro, J. R. & Nakamoto, K. Vibrational spectra of lanthanide hexahalides of the type $(RH)_3LnX_6$. *J. Inorg. Nucl. Chem.* **37,** 1425–1428 (1975).
4. Getsis, A., Balke, B., Felser, C. & Mudring, A. V. Dysprosium-based ionic liquid crystals: thermal, structural, photo- and magnetophysical properties. *Cryst. Growth Des.* **9,** 4429–4437 (2009).
5. Shklyarevskiy, I. O. *et al.* High anisotropy of the field-effect transistor mobility in magnetically aligned discotic liquid-crystalline semiconductors. *J. Am. Chem. Soc.* **127,** 16233–16237 (2005).
6. Micali, N. *et al.* Selection of supramolecular chirality by application of rotational and magnetic forces. *Nature Chem.* **4,** 201–207 (2012).
7. Tishin, A. M. & Spichkin, Y. I. in *The Magnetocaloric Effect and Its Applications* (Institute of Physics Publishing, 2003).
